# Systematic review of economic evaluations for internet- and mobile-based interventions for mental health problems

**DOI:** 10.1038/s41746-022-00702-w

**Published:** 2022-11-23

**Authors:** Fanny Kählke, Claudia Buntrock, Filip Smit, David Daniel Ebert

**Affiliations:** 1 Department of Sport and Health Sciences, Professorship of Psychology and Digital Mental Health Care, Technische Universität München, Munich, Germany; 2 Institute of Social Medicine and Health Systems Research, Medical Faculty, Otto-von-Guericke-University, Magdeburg, Germany; 3 Department of Clinical, Neuro and Developmental Psychology, Amsterdam Public Health Research Institute, Vrije Universiteit, Amsterdam, The Netherlands; 4 Department of Epidemiology and Biostatistics, Amsterdam Public Health Research Institute, Amsterdam University Medical Centers, Location VUmc, Amsterdam, The Netherlands; 5 Centre of Health-Economic Evaluation, Trimbos Institute (Netherlands Institute of Mental Health and Addiction), Utrecht, The Netherlands

**Keywords:** Health care economics, Psychiatric disorders, Quality of life

## Abstract

In view of the staggering disease and economic burden of mental disorders, internet and mobile-based interventions (IMIs) targeting mental disorders have often been touted to be cost-effective; however, available evidence is inconclusive and outdated. This review aimed to provide an overview of the cost-effectiveness of IMIs for mental disorders and symptoms. A systematic search was conducted for trial-based economic evaluations published before 10th May 2021. Electronic databases (including MEDLINE, PsycINFO, CENTRAL, PSYNDEX, and NHS Economic Evaluations Database) were searched for randomized controlled trials examining IMIs targeting mental disorders and symptoms and conducting a full health economic evaluation. Methodological quality and risk of bias were assessed. Cost-effectiveness was assumed at or below £30,000 per quality-adjusted life year gained. Of the 4044 studies, 36 economic evaluations were reviewed. Guided IMIs were likely to be cost-effective in depression and anxiety. The quality of most evaluations was good, albeit with some risks of bias. Heterogeneity across studies was high because of factors such as different costing methods, design, comparison groups, and outcomes used. IMIs for anxiety and depression have potential to be cost-effective. However, more research is needed into unguided (preventive) IMIs with active control conditions (e.g., treatment as usual) and longer time horizon across a wider range of disorders.

**Trial registration:** PROSPERO Registration No. CRD42018093808.

## Introduction

Mental disorders (MDs) are highly prevalent worldwide^1^. Globally, every fifth person is affected, and roughly one-third of adults have experienced mental illness at least once^2^. MDs constitute a substantial burden for individuals and society. Meta-analytic evidence shows an elevated risk of mortality in people with MDs^3,4^ and low quality of life^5^. In addition, MDs appear to be correlated with several physical illnesses^6^ such as stroke, pain, cancer, diabetes mellitus, asthma, heart disease, hypertension, and insomnia^7^. According to the World Health Organization, disease burden as expressed in disability-adjusted life years (DALYs) associated with MDs is substantial and has remained constant over time and across countries^8^. In 2016, Vigo et al. argued that the “true” estimate of the global burden caused by MDs will double compared with earlier estimates and will account for 13% of total DALYs. Hence, the burden of MDs is comparable with those of cardiovascular and circulatory diseases^9^.

MDs are associated with substantial economic costs for society. Associated productivity losses due to absenteeism and presenteeism, earlier retirement, and increased level of healthcare utilization have major influence on society. In 2010, the global costs associated with MDs were estimated at US$2.5 trillion^10^. Indirect costs, such as productivity losses or premature death, were twice as high as direct medical costs related to health service use. In the EU, MD-associated costs are estimated at €798 billion in 2010^11^. However, costs are expected to double by 2030^10^ because of increasing demand and rising costs.

Despite the availability of effective psychological interventions^12^, the majority of individuals with MDs remain untreated^13^ or receive delayed treatment often initiated several years after MD onset^14^. The reasons are multifaceted. Attitudinal barriers, such as low perceived need or a stigma-related desire to handle one’s problems seems to be more important than structural barriers, such as availability of treatment and expenses both for initiating and continuing treatment^15^. One promising approach to overcome these barriers of traditional psychological interventions are internet- and mobile-based interventions (IMIs). IMIs can address these barriers, as IMIs are anonymous, effective, and accessible 24/7^16,17^. Additionally, IMIs can be implemented as stand-alone self-help interventions, as blended care (a face-to-face therapy extended with psychoeducation delivered via the internet) or as part of a stepped care approach in which the amount of support is adjusted to the patient’s needs. IMIs were shown to be effective for treating common MDs across various settings and age groups^18–20^.

Although the initial costs of developing IMIs can be substantial, the low marginal costs of providing IMIs to additional users can result in lower overall expenditure because of an economies of scale effect^16^. However, intervention costs largely vary based on the following four aspects: development phase (new product vs. modified version), scaling-up effects (small vs. large number of users), overestimation of costs (small number of study participants), and efficiency (improving productivity vs. additional costs when newly implemented)^21^. In addition, IMIs are likely to reduce healthcare costs compared with traditional face-to-face treatment, as IMIs reduce costs stemming from therapist’s time and patient’s travel to health services^22^. Hence, IMIs are often touted to be cost-effective despite the weak evidence base for their cost-effectiveness.

Several systematic reviews have attempted to establish the cost-effectiveness of IMIs for MDs in comparison with various control groups. However, the presented evidence on whether IMIs for MDs provide good value for money is inconclusive because some reviews included only few internet-based studies: *n* = 3^23^, *n* = 4^24^, *N* = 12^25^, *n* = 1^26^, and *n* = 5^27^. In addition, 6 of 8 reviews can be considered obsolete today with the latest primary study stemming from 2016^22–25,27,28^, whereas many more studies have since been published, e.g., 26 identified ongoing cost-effectiveness studies for major depression^25^. Moreover, previous reviews used broad definitions of IMIs, e.g., any internet or web enabled platform for diagnosis, screening, treatment, prevention, training, education, or facilitating self-management of MDs^29^. Finally, previous reviews have not always included full health economic evaluations, but have reported costs and effects without relating them to each other^23,29^, and if they did, they only focused on internet-based cognitive behavioral therapy (iCBT)^22^. Likewise, there exist only a few economic evaluations for common treatment options (different types of psychotherapy, pharmacological interventions, such as antidepressants) for depression^30^ and anxiety disorders^24^. Some evidence shows that psychotherapy might be cost-effective compared with pharmacological interventions.

Therefore, a comprehensive overview of the state-of-the-art evidence of IMIs across MDs and symptoms including studies with good methodological quality and full economic evaluations are needed to enable better comparisons and obtain reliable conclusions on guidance, cost perspective, and psychological interventions other than iCBT.

In view of the disease and economic burden of MDs, first, we evaluated whether IMIs for the prevention and treatment of common MDs represent good value for money. Second, we assessed whether these interventions have a good methodological quality. In this respect, our review provides additional evidence to decision makers^31^ to make informed decisions on the allocation of scarce resources to provide sustainable healthcare.

## Results

### Study selection

**Fig. 1:**
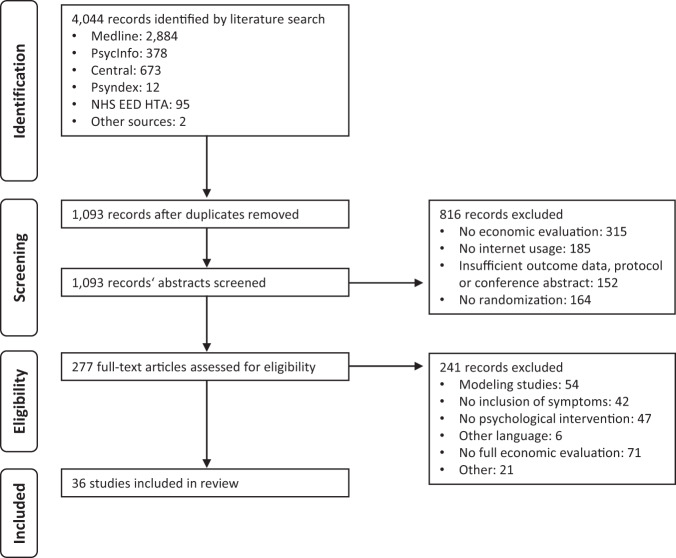
PRISMA flow diagram. Using the Preferred Reporting Items for Systematic Reviews and Meta-Analyses as a screening process, a total of 36 studies were included in the study.

A total of 4044 articles were identified, of which 2951 duplicates and non-relevant studies were removed. Of the 277 full text articles, 36 were eligible for inclusion (Fig. 1), referring to 32 studies. One study was assessed by three articles, and two studies were assessed by two articles. These articles differed by perspectives taken^32–35^, time horizons used^36,37^, or type of analysis^36,38^ used for the evaluation.

### Study characteristics

Table 1 lists relevant study characteristics. Of the 32 studies, 5 have 3 and 1 has 4 comparison groups, whereas 27 only compare 2 groups. In three studies, the same IMI was evaluated^39–42^. The included studies encompassed a total of 10,083 participants. The studies were published between 2010 and 2021 and originated from Australia (*n* = 2), Canada (*n* = 1), Germany (*n* = 7), Netherlands (*n* = 8), United Kingdom (*n* = 6), Spain (*n* = 1), and Sweden (*n* = 10). On average, studies were published in 2015, and most studies were published in 2014 (*n* = 7) and 2017 (*n* = 6). All studies targeted an adult population, except for four studies that were either directed at adolescents (aged 12–19 years, *n* = 2) or people aged >65 years (*n* = 2). Participants were recruited from primary care (*n* = 3481), workplace (*n* = 1260), general population (*n* = 4581), or a mixed setting (*n* = 1057, primary/secondary care and general population). Most of the participants were female (*n* = 7282; 72%) and aged 40 years (mean age 42, SD = 13). The majority of the studies targeted major depressive disorder (MDD) or depressive symptoms (*n* = 15), followed by anxiety disorders (*n* = 7), and obsessive-compulsive disorder (OCD, *n* = 4). Other studies have evaluated sleep disorders (*n* = 2), elevated stress levels (*n* = 2), posttraumatic stress disorder (PTSD, *n* = 1), and suicidal ideation (*n* = 1). Most studies evaluated guided (*n* = 21) or unguided (*n* = 9) interventions, and only two evaluated both guided and unguided IMIs. Most IMIs were based on iCBT (*n* = 35), problem-solving therapy (iPST; *n* = 3), mixed approaches combining different aspects such as problem-solving and emotion regulation (iMA; *n* = 2), positive psychology (iPPI; *n* = 1), and preventive cognitive therapy (iPCT, *n* = 1). On average, an intervention consisted of 7.9 (2–15) sessions and was most often compared with a wait-listed control group (WLC; *n* = 12). Further details of the studies are presented in Table 1.

**Table 1:** Study characteristics.

Study ID	Author ref., country	Disorder target	Setting/sample	Type of evaluation	Sample size (*N*)	Mean age (SD)	Gender (%, female)	Trial arms (*N*)	Delivery period, weeks (sessions)	Guidance	Time horizon
Treatment of subthreshold depression/minor depression/depressive symptoms											
1	Bolier et al.^48^, NL	Depressive symptoms	General population Mild to moderate depressive symptoms (CES-D: 10–24)	CEA	284	43.2 (11.8)	80	iPPI (143) WLC (141)	8 (6)	Unguided	6 months^a^
2	Buntrock et al.^43^, GER	Depressive symptoms	General population Some depressive symptoms (CES-D ≥ 16), but no MDE (SCID-I)	CEA, CUA	406	45 (11.9)	74	iCBT (202) TAU^+^ (204)	6 (6)	Guided	12 months
3	Gerhards et al.^50^, NL	Depressive symptoms	General population At least mild to moderate depressive symptoms (BDI-II score ≥16)	CEA, CUA	303	44.9 (11.6)	57	iCBT (100) iCBT and TAU (100) TAU (103)	8 (8 + 1)	Unguided	12 months
4	Phillips et al.^41^, UK	Depressive symptoms	Workplace, Moderate to severe depressive symptoms, PHQ-9 >scored 2 or more on item 1 and 2 and in total on 5 items	CUA	637	42.5 (9.6)	53	iCBT (318) AC (319)	5 (5)	Unguided	6 weeks
5	Titov et al.^56^, AUS	Depressive symptoms	General population Adults >60 years, “report feeling depressed”	CUA	54	65.4 (3.2)	73	iCBT (29) WLC (25)	8 (5)	Guided	8 weeks
6	Van Luenen et al.^55^, NL	Depressive symptoms	Primary care People with HIV, PHQ-9 score >4 and <20 (mild to moderate symptoms)	CUA	188	46 (10.63)	88	AC + WLC (91) iCBT (97)	8 (8)	Guided	6 months
Treatment of major depression disorder											
7	Brabyn et al.^39^, UK	Depression	Primary care PHQ-9 score ≥10 (cut-off point to detect major depression)	CUA	369	40.6 (13.8)	65	iCBT + uGPC (187) iCBT + uGPC (182)	6 (6)	Guided Unguided	12 months
8	Geraedts et al.^52^, NL	Depression	Workplace At least mild depressive Symptoms (CES-D score ≥16)	CBA, CEA, CUA	231	43.4 (9.0)	62	iPST (116) TAU (115)	6–7 (6)	Guided	12 months
9	Hollinghurst et al.^54^, UK	Depression	Primary care ICD-10 diagnosis of depression (CIS-R), and a BDI score ≥14	CEA, CUA	297	34.9 (11.6)	68	iCBT (149) WLC (148)	16 (10)	Guided	8 months
10	Klein et al.^44^, NL	Recurrent depression	Mixed sample remitted (at least 2 months) individuals with recurrent MDD (last 2 years, SCID-I) and score of ≤10 on HRSD	CEA, CUA	264	46 (10.8)	75	iPCT + TAU (132) TAU (132)	8 (8)	Guided	24 months
11	Littlewood et al.^40^, UK	Depression	Primary care PHQ-9 score ≥10	CUA	691	39.8 (12.6)	67	iCBT 1 + uGPC (210) iCBT 2 + uGPC (242) uGPC (239)	iCBT 1 8 (8) iCBT 2 8 (6)	Unguided Unguided	24 months
12	Nobis et al.^51^, GER	Depression	General population Participants with moderate to severe depressive symptoms (CES-D score ≥23, SCID-I) and diabetes mellitus I or II	CEA, CUA	260	51 (12)	63	iCBT (130) TAU^+^ (130)	6–8 (6–8 + 1)	Guided	6 months
13	Romero-Sanchiz et al.^57^, SP	Depression	Primary care Mild or moderate depressive symptoms (BDI-II score 14–28)	CEA, CUA	296	42.9 (10.3)	76	iCBT (98) iCBT (96) TAU^+^ (102)	10 (10)	Guided Unguided	12 months
14	Warmerdam et al.^53^, NL	Depression	General population Presence of depressive symptoms (CES-D score ≥16)	CEA, CUA	263	45 (12.1)	71	iCBT (88) iPST (88) WLC (87)	8 (8 + 1) 5 (5)	Guided Guided	3 months
15	Yan et al. ^49^, CAN	Depression	Primary care PHQ-9 score ≥10	CUA	1407	47 (17.0)	73	iCBT (415) SC (412) TAU (397) SCP (183)	5(5)	Unguided	12 months^b^
Treatment of anxiety disorders											
16	Bergström et al.^45^, SW	Panic disorder	Mixed sample DSM-IV criteria for panic disorder with or without agoraphobia as primary diagnosis	CEA	113	34.2 (9.5)	62	iCBT (*n* = 53) gCBT (*n* = 60)	10 (10)	Guided	6 months
17	Dear et al.^59^, AUS	Anxiety (GAD)	General population Adults >60 years and difficulties with anxiety (self-report)	CUA	72	65.5 (5.3)	60	iCBT (35) WLC (37)	8 (8)	Guided	8 weeks
18	Nordgren et al.^46^, SW	Anxiety	Primary care DSM-IV, criteria for any anxiety disorder as a primary diagnosis	CEA, CUA	100	35 (13)	63	iCBT (50) AC	10 (7–10)	Guided	10 weeks
19	Hedman et al.^58^, SW	Health anxiety	Mixed sample Primary diagnosis of health anxiety based on diagnostic interview according to DSM-IV	CEA, CUA	81	39.1 (9.7)	74	iCBT (40) AC (41)	12 (12)	Guided	3 months
20	Hedman et al.^60^, SW	Health anxiety	General population Severe health anxiety, diagnostic assessment using MINI	CEA, CUA	158	41.6 (13.4)	79	iCBT (79) iMA (79)	12 (12)	Unguided Unguided	3 months
21, 22 and 23	Hedman et al.^36^^,^^37^, Alaoui et al.^38^, SW	Social anxiety	Mixed sample Diagnostic interview (SCID-I), DSM-IV assessed social anxiety	CEA, CUA, CMA	126	35.4 (11.4)	36	iCBT (64) gCBT (62)	15 (15)	Guided	6 months, 4-year FU
24	Powell et al.^61^, UK	Social anxiety	General Population SPIN-17 score ≥13	CUA	2122	37(13.8)	80	iCBT (1061) WLC (1061), both with access to usual care	6(6)	Unguided	12 months
25	Andersson et al.^63^, SW	OCD	General population Patients after 10 weeks iCBT, meeting the criteria for OCD DSM-IV-TR and Y-BOCS: 12–31	CEA, CUA	101	34 (13.04)	66	iCBT (50) AC (51)	10 (10)	Guided	10 weeks
26	Andersson et al.^64^, SW	OCD	General population Patients after 10 weeks iCBT, meeting the criteria for OCD (DSM-IV-TR) and Y-BOCS: 12–31	CEA	93	36.9 (12.8)	63	iCBT booster session (47) TAU (46)	10 (10)	Guided	2 years^c^
27	Lenhard et al.^65^, SW	OCD	General population Adolescents aged 12–17, moderate to severe symptoms of OCD, CY-BOCS score ≥16)	CEA, CUA	67	14.6 (1.71)	46	iCBT (33) WLC (34)	12 (12)	Guided	3 months
28	Lovell et al.^66^, UK	OCD	Mixed sample Meeting the criteria for OCD (DSM-IV) and moderate to severe symptoms (Y-BOCS score ≥16)	CUA	473	35.86 (12.4)	60	iCBT (157) Self-help workbook^d^ (158) WLC (158)	12 (9)	Guided Guided	3 months^e^
29	Röhr et al.^62^, GER	Posttraumatic Stress	Primary Care Syrian refugees with mild to moderate post-trau-matic stress symptoms (PDS-5 score, range 11–59)	CUA	133	33.0 (11.0)	53	iCBT (65) TAU^+^(68)	4 (2)	Unguided	4 months
Treatment of other disorders											
30	De Bruin et al.^67^, NL	Insomnia	General population Adolescents aged 12–19 years), DSM-IV criteria for insomnia	CEA, CUA	62	15.5 (1.7)	77	iCBT (31) gCBT (31)	6 (6 + 1)	Guided	12 months
31 and 32	Thiart et al.^35^, GER Buntrock et al.^34^, GER	Insomnia	Workplace School teacher with clinically significant insomnia (ISI > 14 and work-related rumination (CI > 14)	CBA, CEA, CUA	128	48.0 (9.9)	74	iCBT (64) + TAU WLC (64) + TAU	6 (6)	Guided	6 months
33 and 34	Ebert et al.^32^, Kählke et al.^33^, GER	Perceived stress	Workplace Employees with elevated symptoms of perceived stress (PSS-10 ≥ 22)	CBA, CEA, CUA	264	43.4 (10.2)	73	iMA (132) WLC (132)	7 (7)	Guided	6 months
35	Lindsäter et al.^68^, SW	Stress-related disorders	General population Adjustment or exhaustion disorder (MINI)	CEA, CUA	100	47 (8.8)	85	iCBT (50) WLC (50)	12 (12)	Guided	3 months
36	Van Spijker et al.^70^, NL	Suicidal ideation	General population Mild to moderate suicidal thoughts (1–26 on BSS)	CEA	236	40.93 (13.7)	66	iCBT (116) WLC^+^ (120)	6 (6)	Unguided	6 weeks^a^

*AC* attention control group, *AUS* Australia, *BA* behavioral activation, *BDI* Beck’s Depression Inventory, *BSS* Beck Scale for Suicide Ideation, *CAN* Canada, *CBA* cost-benefit analysis, *CEA* cost-effectiveness analyses, *CES-D* Center of Epidemiologic Studies Depression Scale, *CIS-R* Clinical Interview Schedule-Revised, *CMA* cost-minimization analysis, *CORE-OM* Clinical Outcomes in Routine Evaluation—Outcome Measure—Generic psychological well-being, *CUA* cost-utility analyses, *CR* cognitive Irritation, *CY-BOCS* Children’s Yale-brown obsessive-compulsive scale, *DSM-IV* Diagnostic and Statistical Manual of Mental Disorders Fourth Edition, *DSM-V* Diagnostic and Statistical Manual of Mental Disorders Fifth Edition, *F2F* face-to-face, *FU* follow-up, *GAD* generalized anxiety disorder, *gCBT* group-administered cognitive-behavioral therapy, *GER* Germany, *HAI* Health Anxiety Inventory, *HRSD* Hamilton Rating Scale for Depression, *iCBT* internet-based cognitive behavioral therapy, *ICD* International Classification of Diseases, *iMA* internet-based interventions applying mixed approaches, *iPPI* internet-based Positive Psychology Intervention, *iPST* internet-based problem-solving therapy, *iPCT* internet-based preventive cognitive therapy, *ISI* Insomnia Severity Index, *LSAS* Liebowitz Social Anxiety Scale, *MDD* major depressive disorder, *MINI* Mini International Neuropsychiatric Interview, *NL* Netherlands, *NR* not reported, *OCD* obsessive-compulsive disorder, *PDS-5* Posttraumatic Diagnostic Scale for DSM-5, *PDSS* Panic Disorder Severity Scale, *PHQ-9* Patient Health Questionnaire-9, *PSS-10* Perceived Stress Scale, *SCID* Structural Clinical Interview for DSM, *SC* Standard Care, *SCP* stepped care pathway, *SCID* Structural Clinical Interview for DSM, *SE* subjective sleep efficiency, *SP* Spain, *SPIN-17* Social Phobia Inventory, *SW* Sweden, *TAU* treatment as usual, *TAU*^*+*^ treatment as usual with access to treatment as usual in a more structured obligatory way (e.g., online website following treatment guidelines, offer the general practitioner (GP) training session following guidelines or informing GP about study and control condition in order to increase usual care), *uGPC* Usual General Practitioner Care, *UK* United Kingdom, *WLC* wait list control condition, *WLC*+ WLC with additional information during the waiting period, *Y-BOCS* Yale-Brown Obsessive-Compulsive Scale.

^a^Costs collected over short period of time and then annualized.

^b^Utility score assessed over 12 weeks were assumed to be consistent at 12 months.

^c^Participants in the IG (Andersson et al.,^63^) received an iCBT treatment over 10 weeks, the CG received the iCBT after the 10 weeks (crossover). After a 4-month follow-up half of the participants were randomized to receive an additional booster session, 8 and 20 months after randomization to booster session.

^d^Guided self-help consisted of a self-help book: Overcoming OCD: A Workbook, written by the trial team. Participants received weekly guidance from a PWP for one initial session of 60 min (either face to face or by telephone, depending on patient preference) followed by up to ten 30-min sessions over a 12-week period.

^e^After 3 months all three groups had access to high intensity CBT (f2f), therefore only the 3 months’ time point was reported.

Most studies (*n* = 16) conducted both a cost-effectiveness analysis (CEA) and a cost-utility analysis (CUA). Other studies focused solely on either CUAs (*n* = 10) or CEAs (*n* = 4). Three studies conducted a cost-benefit analysis (CBA) in addition to CEA and CUA. The included studies differed in perspectives taken: societal (*n* = 15), healthcare (*n* = 6), and both perspectives (*n* = 9). In the remaining studies, the employer’s perspective (*n* = 3) alone or in combination with other perspectives were applied. One study conducted a cost-minimization analysis (CMA). Three studies did not report the study perspective. The time horizon of the follow-ups varied across studies ranging from ≤3 months (*n* = 12), >3 to ≤6 months (*n* = 8), >6 to ≤12 months (*n* = 9) to 2 years (*n* = 4).

### Quality assessment

Table 2 contains the Consensus on Health Economic Criteria (CHEC) quality scores. The quality of studies was mainly good (average total score = 85%, range 56–100%). Three studies met all CHEC criteria^34,43,44^, whereas three studies showed average quality^41,45,46^. Common reasons for the lower quality were the lack of reporting on the generalizability of the results (*n* = 29), an insufficient time horizon (*n* = 16), or lack of sensitivity analyses (*n* = 8). All studies met the items on appropriateness of the economic study designs and outcome measurement.

**Table 2:** CHEC quality assessment.

Item		Study ID																	
		1	2	3	4	5	6	7	8	9	10	11	12	13	14	15	16	17	18
**(a) (articles 1–18)**																			
1	Is the study population clearly described?	1	1	1	1	1	1	1	1	1	1	1	1	1	1	1	1	1	1
2	Are competing alternatives clearly described	1	1	1	0	1	1	1	1	1	1	1	1	1	1	1	1	1	1
3	Is a well-defined research question posed in answerable form?	1	1	1	0	1	1	1	1	1	1	1	1	1	1	1	1	1	0.5
4	Is the economic study design appropriate to the stated objective?	1	1	1	1	1	1	1	1	1	1	1	1	1	1	1	1	1	1
6	Is the chosen time horizon appropriate include relevant costs and consequences?	0.5	1	1	0	0	1	1	1	1	1	1	0.5	1	0	1	1	0	0
7	Is the actual perspective chosen appropriate?	1	1	1	0	1	1	1	1	1	1	1	1	1	1	0.5	0	0	0
8	Are all important and relevant costs for each alternative identified?	1	1	1	1	1	1	1	1	1	1	1	1	1	1	1	0	1	1
9	Are all costs measured appropriately in physical units?	1	1	1	1	1	1	1	1	1	1	1	1	1	1	1	1	1	1
10	Are costs valued appropriately?	1	1	1	0	0.5	1	1	1	1	1	1	1	1	1	1	0	1	0
11	Are all important and relevant outcomes for each alternative identified?	1	1	1	1	1	1	1	1	1	1	1	1	1	1	1	1	1	1
12	Are all outcomes measured appropriately?	1	1	1	1	1	1	1	1	1	1	1	1	1	1	1	1	1	1
13	Are outcomes valued appropriately?	-	1	1	1	1	1	1	1	1	1	1	1	1	1	0	-	1	1
14	Is an incremental analysis of costs and outcomes of alternatives performed?	1	1	0	0	1	1	1	1	1	1	1	1	1	1	1	1	1	1
15	Are all future costs and outcomes discounted appropriately?	-	-	-	-	-	-	-	-	-	1	1	-	-	-	-	-	-	-
16	Are all important variables, whose values are uncertain, appropriately subjected to sensitivity analysis?	1	1	1	0	0.5	1	1	1	0.5	1	1	1	0	1	1	0.5	0.5	1
17	Do the conclusions follow from the reported data?	1	1	1	1	1	1	0.5	1	1	1	1	0.5	0	1	1	1	1	1
18	Does the study discuss the generalizability of the results to other settings and patient/client groups?	0.5	1	0.5	0	0	0	0	0	0.5	1	0.5	0	0.5	0	0	0	0.5	0
19	Does the article indicate that there is no potential conflict of interest of study researcher(s) and funder(s)?	1	1	1	1	1	1	1	1	0.5	1	1	1	1	1	1	1	1	0.5
20	Are ethical and distributional issues discussed appropriately?	1	1	0	0	0	1	0	0	0.5	1	0	0	0	0	1	0	0	0
Score %^a^		94	100	86	56	78	94	86	89	89	100	92	83	81	83	86	68	78	67
**(b) (articles 19–36)**																			
		19	20	21	22	23	24	25	26	27	28	29	30	31	32	33	34	35	36
1	Is the study population clearly described?	1	1	1	1	1	1	1	1	1	1	1	1	1	1	1	1	1	1
2	Are competing alternatives clearly described	1	1	1	1	1	1	0,5	1	1	1	1	1	1	1	1	1	1	1
3	Is a well-defined research question posed in answerable form?	0.5	1	1	1	1	0.5	0.5	0.5	1	1	1	1	1	1	1	1	1	1
4	Is the economic study design appropriate to the stated objective?	1	1	1	1	1	1	1	1	1	1	1	1	1	1	1	1	1	1
6	Is the chosen time horizon appropriate include relevant costs and consequences?	0	0	0.5	1	1	1	0	1	0	0.5	1	1	1	1	0.5	0.5	0.5	0
7	Is the actual perspective chosen appropriate?	1	1	1	1	1	1	1	1	1	1	1	1	1	1	1	1	1	1
8	Are all important and relevant costs for each alternative identified?	1	1	1	1	1	1	1	1	1	1	1	1	1	1	1	1	1	1
9	Are all costs measured appropriately in physical units?	1	1	1	1	1	1	1	1	1	1	1	1	1	1	1	1	1	1
10	Are costs valued appropriately?	1	1	1	0	1	1	1	0.5	1	1	0.5	1	1	1	1	1	1	1
11	Are all important and relevant outcomes for each alternative identified?	1	1	1	1	1	1	1	1	1	1	1	1	1	1	1	1	1	1
12	Are all outcomes measured appropriately?	1	1	1	1	1	1	1	1	1	1	1	1	1	1	1	1	1	1
13	Are outcomes valued appropriately?	1	1	1	1	1	1	1	-	1	1	1	1	-	1	-	1	1	-
14	Is an incremental analysis of costs and outcomes of alternatives performed?	1	1	1	1	1	1	1	1	1	1	1	1	1	1	1	1	1	1
15	Are all future costs and outcomes discounted appropriately?	-	-	-	0	1	-	-	0	-	-	-	-	-	-	-	-	-	-
16	Are all important variables, whose values are uncertain, appropriately subjected to sensitivity analysis?	1	0.5	1	1	1	0	0.5	0.5	1	1	0	1	1	1	1	1	1	1
17	Do the conclusions follow from the reported data?	1	0	1	1	1	1	0.5	1	1	1	1	1	1	1	1	1	1	1
18	Does the study discuss the generalizability of the results to other settings and patient/client groups?	1	0.5	0.5	0	0.5	0	1	0.5	0.5	0.5	0.5	0	0.5	1	0	0	1	0.5
19	Does the article indicate that there is no potential conflict of interest of study researcher(s) and funder(s)?	1	1	1	1	1	1	0	1	1	1	1	1	1	1	1	1	1	1
20	Are ethical and distributional issues discussed appropriately?	0	0	0.5	0	0	1	0	0	0.5	0.5	1	0	0	1	0	0	1	0.5
Score %^a^		86	78	92	79	92	86	72	78	89	92	89	89	91	100	85	86	97	88

The item number 5 of the CHEClist is excluded because it only applies to modeling studies. 0 no, 0.5 suboptimal, 1 yes.

Score expressed as percentage of the maximum score.

**Fig. 2:**
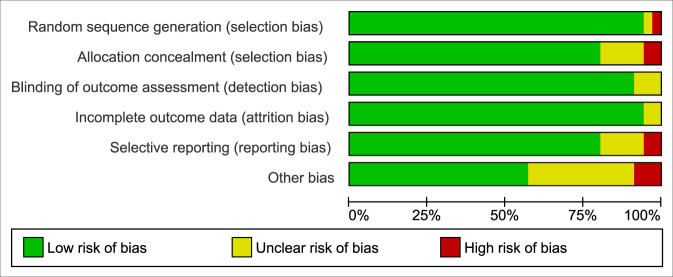
Risk of bias assessment. The graph displays the authors’ judgments on risk of bias of each included study, presented as percentage totals according to the Cochrane Collaborations tool.

Regarding risk of bias (RoB), most studies showed good (*n* = 22), and only a few studies showed fair (*n* = 10) or poor (*n* = 4) quality (Fig. 2 and Table 3). Detection, attrition, and selection bias were low. By contrast, reporting bias (*n* = 9) and other biases were high (*n* = 14). Selective reporting may arise when outcomes for a CEA are not sufficiently described in study protocols and outcome paper. Other biases may arise when there are insufficient information or limitations because of the high complexity of assessing outcomes, e.g., the annualization of short term costs. The agreement for CHEC and RoB between the two raters with Cohen’s kappa (*κ*) = 0.90–0.91 can be considered almost perfect^47^.

**Table 3:** Risk of bias assessment.

Nr	Author (ref.)	Random sequence generation (selection bias)	Allocation concealment (selection bias)	Blinding of outcome assessment (detection bias)	Incomplete outcome data (attrition bias)	Selective reporting (reporting bias)	Other bias
1	Bolier et al.^48^	Low risk	Unclear risk	Low risk	Low risk	Low risk	Unclear risk
2	Buntrock et al.^43^	Low risk	Low risk	Low risk	Low risk	Low risk	Low risk
3	Gerhards et al.^50^	Unclear risk	Low risk	Low risk	Low risk	Low risk	Low risk
4	Phillips et al.^41^	Low risk	Low risk	Low risk	High risk	High risk	High risk
5	Titov et al.^56^	Low risk	Low risk	Low risk	Low risk	Low risk	High risk
6	Van Luenen et al.^55^	Low risk	Low risk	Unclear risk	Low risk	Low risk	Unclear risk
7	Brabyn et al.^39^	Low risk	High risk	Low risk	Low risk	Low risk	Unclear risk
8	Geraedts et al.^52^	Low risk	Low risk	Low risk	Low risk	Low risk	Low risk
9	Hollinghurst et al.^54^	Low risk	Low risk	Unclear risk	Low risk	Low risk	Unclear risk
10	Klein et al.^44^	Low risk	Low risk	Low risk	Low risk	Low risk	Low risk
11	Littlewood et al.^40^	Low risk	Low risk	Low risk	Low risk	Low risk	Low risk
12	Nobis et al.^51^	Low risk	Unclear risk	Unclear risk	Low risk	Low risk	Unclear risk
13	Romero-Sanchiz et al.^57^	Low risk	Low risk	Low risk	Low risk	Low risk	Low risk
14	Warmerdam et al.^53^	Low risk	Low risk	Low risk	Low risk	Low risk	Low risk
15	Yan et al.^49^	Low risk	Low risk	Low risk	Low risk	Low risk	Unclear risk
16	Bergström et al.^45^	Low risk	Unclear risk	Low risk	Unclear risk	High risk	Low risk
17	Dear et al.^59^	Low risk	Low risk	Low risk	Low risk	Unclear risk	Unclear risk
18	Nordgren et al.^46^	Low risk	Low risk	Low risk	Low risk	Unclear risk	Low risk
19	Hedman et al.^58^	Low risk	Low risk	Low risk	Low risk	Unclear risk	Unclear risk
20	Hedman et al.^60^	Low risk	Low risk	Low risk	Low risk	Low risk	Low risk
21	Hedman et al.^36^	Low risk	Low risk	Low risk	Low risk	Unclear risk	Low risk
22	Hedman et al.^37^	Low risk	Low risk	Low risk	Low risk	Unclear risk	Low risk
23	Alaoui et al.^38^, SW	Low risk	Low risk	Low risk	Low risk	Low risk	Low risk
24	Powell et al.^61^	Low risk	Low risk	Low risk	Low risk	Low risk	Unclear risk
25	Andersson et al.^63^	Low risk	Low risk	Low risk	Low risk	Unclear risk	Low risk
26	Andersson et al.^64^	High risk	High risk	Low risk	Unclear risk	Low risk	Low risk
27	Lenhard et al.^65^	Low risk	Unclear risk	Low risk	Low risk	Low risk	Unclear risk
28	Lovell et al.^66^	Low risk	Low risk	Low risk	Low risk	Low risk	Low risk
29	Röhr et al.^62^	Low risk	Low risk	Low risk	Low risk	Low risk	Unclear risk
30	De Bruin et al.^67^	Low risk	Unclear risk	Low risk	Low risk	Low risk	Low risk
31	Thiart et al.^35^	Low risk	Low risk	Low risk	Low risk	Low risk	Unclear risk
32	Buntrock et al.^34^	Low risk	Low risk	Low risk	Low risk	Low risk	Low risk
33	Ebert et al.^32^	Low risk	Low risk	Low risk	Low risk	Low risk	Low risk
34	Kählke et al.^33^	Low risk	Low risk	Low risk	Low risk	Low risk	Low risk
35	Lindsäter et al.^68^	Low risk	Low risk	Low risk	Low risk	Low risk	Low risk
36	Van Spijker et al.^70^	Low risk	Low risk	Low risk	Low risk	Low risk	High risk

### Findings of included studies

Supplementary Table 1 displays the following characteristics and outcomes for each of the included health economic evaluations: perspective taken, cost categories used, type of health outcome and measurements, mean incremental cost-effectiveness ratio (ICER) or cost-utility ratio (ICUR) and its position in the quadrant of the cost-effectiveness plane, and probabilities of the intervention being cost-effective given various willingness to pay (WTP) thresholds. This table lists all costs in national currency units and for the index year as published by the primary studies. In the next section, probabilities are only listed if reported in the studies: CUA, WTP threshold of £30.000 per QALY gained; CEA, WTP of £0 per additional, e.g., treatment responder.

### MDD

#### Treatment of MDD, minor/subthreshold depression, and depressive symptoms

Fifteen studies evaluated IMIs for MDD (*n* = 8) and depressive symptoms (*n* = 5), whereas two studies focused on depression onset and relapse prevention. The control conditions consisted of alternative guidance formats: iPST, iPPI, iPCT, standard care, stepped care pathway, treatment as usual (TAU), WLC, and attention control (AC). Depressive symptom severity at baseline had no recognizable effect on cost-effectiveness.

One-third of the studies (*n* = 5) evaluated unguided IMIs based on CBT (*n* = 4) or positive psychology (*n* = 1^48^). As for unguided IMIs compared with TAU (*n* = 3), results from the CUA conducted from the healthcare perspective after 1–2 years did not suggest an economic merit^40,49^ (at a WTP threshold of £30,000, the probability of cost-effectiveness varied: CUA = 4–38%). However, findings from the societal perspective suggested that one IMI^50^ had an acceptable likelihood of being cost-effective (at WTP = 0, CEA = 70%; at WTP = £30,000; CUA = 55%). Compared with WLC or AC (*n* = 2), unguided IMIs from the societal perspective provided only little and unclear evidence for cost-effectiveness (at the WTP = 0, CEA = 20%^48^; CUA was not reported^41^).

Six of the 15 studies evaluated guided IMIs based on iCBT (*n* = 4) or iPST (*n* = 2). Two guided IMIs were compared with TAU and showed opposing results after 6–12 months. Findings from the societal perspective showed a moderate-to-acceptable likelihood of being cost-effective (at WTP = 0, CEA = 48^51^–62%^52^), one above^52^ and one below^51^ the proposed threshold of £30,000. From the employer’s perspective, one IMI was the dominant treatment option (WTP = 0, CEA = 55%)^52^.

Four guided^53–56^ IMIs, compared with WLC, were considered cost-effective (<£30,000 per QALY gained, probabilities ranging from 55^53^ to 98%^55^) from the societal and healthcare perspective. Results of the cost-effectiveness analyses were unclear^54^ or showed a low likelihood of being cost-effective at a WTP of nil from a societal perspective (CEA = 30–38%^53^).

Two studies compared similarly effective guided to unguided IMIs after 12 months. In one study, from the societal perspective, both IMIs generated less costs than usual care and were judged cost-effective^57^ (<£30,000 per QALY gained, probabilities were not reported). In the other study, from the NHS’ perspective, the guided IMI resulted in more QALYs gained at lower costs than the unguided IMI (considered cost-effective, at WTP = £ 30,000, CUA = 55%^39^).

#### Prevention of MDD onset and relapse prevention

The remaining studies evaluating guided IMIs (*n* = 2) focused on the prevention^43^ or relapse^44^ of MDD in comparison with usual care. Findings from cost-effectiveness analyses employing a societal perspective suggested a moderate likelihood of them being cost-effective, with probabilities ranging from 38% to 40% at a WTP of nil. CUA showed a moderate (CUA = 40%^44^) to acceptable (CUA = 60%^43^; ICUR < £30,000 per QALY gained) likelihood of them being cost-effective. From the healthcare perspective, one IMI^43^ showed a small likelihood of being cost-effective per depression-free year gained (WTP = 0, CEA = 17%) but was considered cost-effective when below the cost-utility threshold (at WTP = £30,000, CUA = 64%).

#### Anxiety disorders or symptoms

Eight studies evaluated guided (*n* = 5) and unguided (*n* = 3) IMIs for anxiety disorders based on CBT compared with TAU, AC, WLC, group-administered CBT (gCBT), or iMA. The included studies targeted panic disorder (*n* = 1), generalized anxiety disorder (GAD) (*n* = 1), health anxiety (*n* = 2), social anxiety (*n* = 2), any anxiety disorder (*n* = 1), and PTSD (*n* = 1).

Three studies comparing guided IMIs to AC or WLC in the short term (8–12 weeks) were judged cost-effective from the societal and healthcare perspectives (<£30,000, per QALY gained, probabilities >90%^46,58,59^). Cost-effectiveness analyses showed that the IMIs dominated the control group by generating less costs at higher effects from the societal perspective (at WTP = 0, CEA = 64^58^–95%^46^).

Two studies comparing guided IMIs with gCBT after 6 months to 4 years provided good evidence for their cost-effectiveness. The first IMI was cost-effective from the societal perspective in the short and long term (<£30,000 per QALY gained, CUA = 34^37^–79%^36^). Results of the cost-effectiveness analyses showed that the IMI produced less costs at higher effects (WTP = 0, CEA = 81%^36^) in the short term and increased costs with lower probability of being cost-effective in the long term (WTP = 0, CEA = 62%^37^). From a healthcare perspective, the same IMI was cost-effective based on a CMA (WTP = £30,000, CMA = 67%^38^). The second IMI was likewise cost-effective from the healthcare perspective, being the dominant treatment option (WTP = 0, CEA = 75%^45^).

By contrast, for two studies evaluating unguided IMIs, the results of the cost-utility analyses were considered cost-effective (yet no probabilities were reported), but the CEA did not support these findings. The first IMI^60^ was compared with unguided iMA from a societal perspective, which resulted in higher costs per responder, showing low probabilities of being cost-effective (at WTP = 0, CEA = 8%), but being below the £30,000 threshold per QALY gained. The second IMI generated less costs per QALY gained than WLC from both healthcare and societal perspectives^61^. A third unguided study compared an unguided IMI (self-help app) targeting posttraumatic stress^62^ with TAU from a healthcare perspective and showed a low probability of cost-effectiveness (≈27% at WTP = £30,000 per QALY gained).

### OCD

Three studies evaluated guided IMIs for OCD based on CBT in comparison with either self-help book with guidance, WLC, AC, or a booster session. The evidence for cost-effectiveness was contradictory regarding QALYs and moderate regarding clinical outcomes because of heterogeneous control conditions.

From the societal and healthcare perspective, one IMI was cost-effective compared with AC being below the acceptable threshold per QALY gained (at WTP £30,000, CUA = 90–95%^63^). By contrast, the IMI was judged not cost-effective per additional remission in the short term (at WTP = 0, CEA = 0–15%) nor per relapse prevented after 2 years when a booster session was offered in a crossover design (at WTP = 0, CEA = 0–18%^64^).

Two studies compared IMIs with WLC after 3 months. From the societal and healthcare perspectives, one study did not report probabilities of cost-effectiveness nor ICUR^65^, and the other was neither cost-effective compared with WLC (ICUR > 30,000 per QALY gained^66^, CUA = 35–52%) nor more effective than guided self-help.

#### Other mental disorders

Most of the remaining five studies used CBT (guided, *n* = 4; unguided, *n* = 1), and only one intervention used iMA. The IMIs targeted insomnia, perceived stress or stress-related disorders, or suicidal ideation and showed a moderate to high probability of cost-effectiveness.

IMIs targeting insomnia were cost-effective per QALY gained but unconvincing regarding cost-effectiveness analyses. One IMI was cost-effective compared with WLC and below the threshold per QALY gained (at WTP = £30,000^34^, CUA = 99%) from the societal and healthcare perspectives. Cost-effectiveness analyses also showed a high probability of being cost-effective, dominating the WLC per additional treatment responder (CEA = 87%, employer’s perspective^35^) or symptom-free status (CEA = 94%, societal perspective^34^), but generating higher costs from the healthcare perspective, leading to a low probability of cost-effectiveness (CEA = 6%^34^).

Another IMI^67^ was compared with gCBT from a societal perspective. Both treatments showed similar effects, and the IMI led to a high probability of cost-savings while trading off health gains (at WTP = 0, CEA = 95%) but generating more QALYs (at WTP = £30.000, CUA = not reported).

IMIs targeting adjustment or exhaustion disorder, or perceived stress, were mostly cost-effective compared with WLC. Based on findings of the cost-utility analyses, two IMIs were below the threshold of £30.000, showing high probabilities of being cost-effective from the societal perspective (CUA = 75^68^–79%^33^). In addition, findings of the cost-effectiveness analyses showed that both IMIs dominated the WLC, yielding acceptable probabilities of cost-effectiveness at a WTP of nil from the employer’s (CEA = 67%^69^) and societal (CEA = 70%^33^) perspectives, but not from the healthcare perspective (CEA = 12%^68^) where higher costs were generated.

The only unguided IMI^70^ targeting suicidal ideation dominated the WLC, generating a high probability of being cost-effective at a WTP of nil from the societal perspective (CEA = 92%).

#### Workplace setting

Cost-benefit analyses evaluating costs relevant to the employer yielded a benefit-to-cost ratio (BCR) > 1 (1.6–3.1) and net-benefit greater zero (181–417), which indicates that guided IMIs were cost-effective when compared with TAU and WLC for the treatment of insomnia^35^, elevated stress^69^, and depression^52^.

#### Guidance and comparators

The majority of studies evaluated guided IMIs (*n* = 24), which were mostly cost-effective, indicated by ICURs < £30,000/QALY gained, irrespective of the types of control conditions. However, unguided IMIs (*n* = 11) showed little evidence of cost-effectiveness.

## Discussion

This review presents a comprehensive overview of trial-based economic evaluations providing evidence regarding the cost-effectiveness of IMIs for the prevention and treatment of MDs and symptoms. This review identified 32 studies applying societal (*n* = 24), healthcare (*n* = 15), and employer’s perspectives (*n* = 3) in 65 full economic evaluations (CBA, *n* = 3; CEA, *n* = 31; CMA, *n* = 1; CUA, *n* = 30).

In half of the CEAs (*N* = 14; MDD, *n* = 3; anxiety, *n* = 5; stress, *n* = 3; sleep *n* = 2; suicidal ideation, *n* = 1), the IMI was the dominant treatment option, which means that more health effects were generated at lower costs in comparison with control conditions. Of these, two did not report a WTP and five showed a high probability (≥80%) of being more cost-effective than control conditions at a WTP of nil. For all CEAs, the range of probability at WTP of nil varied from 0 to 95%. Regarding cost-utility, most interventions were cost-effective, being either dominant (*n* = 13) and/or below the WTP threshold of £30,000 per QALY gained (*n* = 26) compared with any control condition and often regardless of the perspectives taken. By applying the criterion that an IMI showed at least an 80% probability of cost-effectiveness at WTP of £30,000 compared with a control condition (if reported), 11 IMIs were judged to be cost-effective. Cost-benefit analyses from the employer’s perspective (*n* = 3) yielded positive net benefits representing the money gained after costs were recovered. In addition, the overall quality of studies (CHEC) was good (*n* = 30), only a few were excellent (*n* = 3) or average (*n* = 3). Reasons for a low rating were no discussion of generalizability, short time horizon, or lack of sensitivity analyses. Regarding RoB, most studies showed good quality (*n* = 22), and only few studies (*n* = 6) showed at least one item at high risk of bias.

Our findings expand and strengthen the evidence base for the cost-effectiveness of IMIs. First, our findings support the evidence of cost-effectiveness of guided IMIs for depression and anxiety^24,25,27–29^. Second, our review includes new evidence related to under-researched disorders such as OCD (*n* = 4), PTSD (*n* = 1), stress (*n* = 3), and sleep (*n* = 2). However, given the limited number of studies, more evidence is needed.

The strength of this review is related to the comprehensive and systematic search strategy in several electronic databases for common MDs and problems, and the resulting health-economic comparisons. The quality of studies was assessed on the methodology of cost-effectiveness analyses and RoB. To further improve comparability and clarity, economic outcomes were converted to Pound Sterling for the reference year 2020 and mapped to the quadrant of the cost-effectiveness plane in which the mean ICER fell (as far as reported in the primary studies). Likewise, unified thresholds and transparent criteria proposed by the authors were used.

However, the comparability of evidence across the studies was hampered by the high heterogeneity stemming from different study designs, methods, study populations, outcome measures, time horizons, comparators, economic perspectives, cost items, and their evaluation. As a case in point, the operationalization of societal costs and intervention costs varied widely. The costs of development and maintenance of the IMIs were often not included or incompletely reported, leading to a possible underestimation of intervention costs. Half of the studies (*n* = 16) did not report intervention costs or only valued the time for the therapist needed to support the participants.

Another limitation is the lack of interpretability regarding cost-effectiveness, as the WTP for diagnosis-specific measures (e.g., symptom-free, reliable change) is unknown and the WTP threshold for QALYs is somewhat arbitrary, as universally accepted thresholds are unavailable^71^. For healthcare decision-making, several countries compared ICER to a reference value (generic cost-effectiveness threshold) that represents the maximum cost the health system is willing to pay for a health outcome. These generic thresholds vary largely depending on the methods (e.g., per capita income, benchmarking interventions, and leagues tables: ranking the ICERs of interventions given a specific budget) and setting^71^. An international survey assessing the individual WTP for one additional QALY gained showed that the thresholds vary between countries (e.g., Taiwan 2.14 times the UK’s per QALY gained)^72^. Consequently, higher thresholds lead to interventions being adopted earlier than in countries with lower thresholds. Beyond the narrow cost-effectiveness arguments, other criteria of health technology assessment should also be considered for decision-making purposes (e.g., disease burden, prognosis, medical ethics, access, equity, feasibility of implementation and scale-up of the interventions, and acceptability of the intervention by its intended recipients)^73^. Furthermore, most health-economic evaluations alongside randomized controlled trials (RCTs) are not powered to detect differences in costs nor QALYs. This might result in non-significant differences in costs and QALYs, which can lead to wider uncertainty intervals surrounding the ICER estimates^74^. Moreover, some studies (*n* = 3) only collected data over a short period of the study duration and annualized effects and costs. In addition, in some studies (*n* = 6), the uncertainty surrounding the ICER point estimates was not clear because neither the CEA plane nor the cost-effectiveness acceptability curve where reported. As all studies were conducted in Western countries, especially in the NW Europe, the generalizability of results is restricted to these regions. In this regard, selection bias could have been introduced, as only studies published in German and English were included.

The results may lead to several clinical implications. The review could be important for decision-makers when allocating scant resources to meet the demands for the many in need of sustainable healthcare. With the increasing use of economic data in decision-making in public mental health and the increasing societal and economic burden of MDs, consideration of the cost-effectiveness of psychological preventive interventions and treatments is becoming increasingly important. IMIs might be an important way forward. Moreover, since the COVID-19 pandemic, increasing numbers of patients and health services had to shift toward IMIs for the receipt and delivery of mental healthcare. Thus, this may have paved the way for scaled-up uptake of IMIs.

Despite the high heterogeneity stemming from intervention types and comparators of the included studies, some promising trends toward specific mental health targets were seen. Recommendations for policy makers and relevant stakeholders can be made, relating to existing NHS guidelines^75^ for the application of low-intensity psychosocial interventions in depression and anxiety. Based on our results, guided IMIs for MDD and anxiety disorders should be offered as treatment option. The evidence regarding the cost-effectiveness of under-researched disorders (e.g., OCD, sleep, and stress) and of unguided interventions is limited, and offering such interventions should rely on case-by-case decisions. However, unguided IMIs are scalable and easy to implement, showing a high potential to make an impact at a population level.

Besides these recommendations related to financial aspects, the implementation setting, target population, symptom severity and disorders should be considered. In addition, knowledge about diverse stakeholders’ views and values relevant to priority setting enables decision-makers to make better-informed decisions and appropriate judgments about allocation of scant resources.

In practice, most healthcare providers are receptive to the advantages of IMIs as part of their treatment. However, IMIs should meet the criteria of government reimbursement mechanisms, like the National Institute for Health and Clinical Excellence’s (NICE) in the UK or the one for digital health applications in medical and psychotherapeutic care in Germany, to become sustainable. Such criteria include evidence on effectiveness, interoperability, safety, and data security^76^.

Following this, we provide several recommendations for future research. First, various anxiety disorders such as panic disorder, GAD, and social anxiety were underrepresented, and disorders such as specific phobias were not found for this review. Moreover, studies were only conducted in resource-rich high-income countries. Hence, we recommend focusing on under-researched disorders and conducting research in low- and middle-income countries.

Second, we recommend publishing study protocols that adhere to economic evaluation guidelines (ISPOR^77^ and CHEERS^78^) and quality checklists (Drummond^31^ and CHEC^79^), thereby minimizing biases and improving study quality (e.g., reporting of uncertainty, sensitivity analysis and combined reporting of disease-specific and generic health outcomes to facilitate comparability, and interpretation for decision-making).

Third, the cost-effectiveness of IMIs for MDs and symptoms was frequently based on short term findings (6–16 weeks, *n* = 13), whereas the remaining studies reported findings based on moderate (6–12 months, *n* = 14) to long follow-up periods (2–4 years, *n* = 3). We recommend conducting economic evaluations over longer follow-up periods to better capture longer-term productivity losses and gains, especially in preventive interventions in remittent disorders, such as anxiety disorders.

Fourth, more research is needed on IMIs compared with active control condition across all disorders to establish the cost-effectiveness of IMIs as possible alternative to face-to-face treatments.

Fifth, studies are needed to carefully choose the perspectives taken depending on the decision maker, target population, disorder, or setting. For employers, productivity losses are most important, whereas from a healthcare system’s perspective, a high healthcare coverage for people affected by disorders is prioritized.

Finally, the acceptability of an IMI among patients and relevant stakeholders is worth investigating to provide more insights pertinent for the implementation, uptake, and use thereof.

In conclusion, this systematic review provides an overview of economic evaluations of internet-based interventions for the treatment and prevention of MDs. Guided iCBTs for anxiety disorders and MDD showed a high probability of being cost-effective. IMIs for insomnia, suicidal ideation, and stress had the potential of being cost-effective, whereas the evidence base for the cost-effectiveness of IMIs in OCD was not very firm. Although many studies were identified, more robust conclusions about the cost-effectiveness of IMIs could not be reached given the high heterogeneity across the studies with regard to methodologies, interventions, and comparators in a range of disorders and symptoms among various populations and age groups. More cost-effectiveness research is warranted in unguided and preventive IMIs that are proven to be effective, specifically in under-researched disorders and symptoms and preferably over longer time horizons. From a methodological perspective, future studies should more stringently adhere to existing health-economic guidelines to increase comparability and enhance their value for decision-making purposes in healthcare.

## Methods

The guidelines of Preferred Reporting Items for Systematic Reviews and Meta-Analyses^80^ and preparation for systematic reviews of economic evaluations^81^ were followed. This systematic review was registered in the international prospective register of systematic reviews, PROSPERO (CRD42018093808^82^).

### Search strategy

An extensive literature search was conducted, using the following electronic databases: MEDLINE, PsycINFO, Cochrane Central Register of Controlled Trials (CENTRAL), PSYNDEX, and National Health Service (NHS) Economic Evaluations Database. Relevant articles published before 10/05/2021 were identified using standardized subject terms. A search strategy consisting of four main categories was applied for each database selecting articles referring to (1) intervention, treatment, prevention, or psychotherapy; (2) MDs, (3) internet, online, or mobile-based; and (4) economic evaluation (Supplementary Table 2).

### Eligibility criteria

Studies were eligible for inclusion if they met the following inclusion criteria:

Population: participants regardless of age with a diagnosis of MD or symptoms such as MDD, dysthymia, bipolar disorder, social phobia, panic disorder, GAD, PTSD, OCD, specific phobia, and separation anxiety, sleep disorders, or transdiagnostic key symptoms such as suicidal thoughts, and psychological distress, all of which were required to be assessed with validated self-report questionnaires or being based on diagnostic interviews.

Intervention: psychological interventions that are provided in an online setting, defined as internet-, online-, web-, or mobile-based and grounded in CBT, interpersonal therapy, problem-solving therapy, positive psychology intervention, psychodynamic therapies, behavior therapy or behavior modification, systemic therapies, third-wave cognitive behavioral therapies, humanistic therapies, or integrative therapies. Internet-based interventions can be “guided”, offering patients human support by a psychotherapist via email or chat or automated feedback delivery, or “unguided”, only offering self-help interventions without any additional human support.

Comparator: included one of the following control groups: another psychological intervention, TAU, WLC, or AC group.

Outcome measures: reported economic evaluation estimates based on CEA, CUA, CBA, and CMA of a full economic evaluation, which means that the study compared both costs and effects (e.g., QALYs, treatment response, relapse avoided, and remission) of two or more alternatives.

Study types: RCTs, full texts are accessible as peer-reviewed papers, in English or German.

Studies were excluded if the intervention was not delivered online. IMIs were excluded when provided in combination with a face-to-face or video-based sessions delivered by a therapist (i.e., blended intervention). Studies were excluded if they did not report a meaningful outcome measure for economic evaluation (e.g., point improvement on an ordinal scale). Health-economic modeling studies were excluded because of methodological differences compared with trial-based economic evaluations (e.g., not directly based on observational data) limiting internal validity of the review. Conference abstracts, protocol papers, non-peer-reviewed papers, cost of illness, observational studies, cohort studies, case studies, pilot studies, and feasibility studies were also excluded.

### Study selection and extraction

First, titles and abstracts of the identified articles were screened. Then, studies were evaluated whether they met the criteria in full text by two independent researchers, F.K. and C.B. Disagreement was discussed and/or a third reviewer (D.D.E.) consulted. Interrater agreement (Cohen’s kappa) of the two reviewers was examined.

Data of eligible studies were extracted using the Consolidated Health Economic Evaluation Reporting Standards Checklist^78^: (1) characteristics of participants (setting, age, sex, and screened symptoms/diagnosis), (2) study design (sample size, trial arms, and assessment points), (3) intervention (psychological approach, guidance, and length of intervention), (4) economic outcome measures, (5) type of economic evaluation, (6) characteristics of derived costs (cost categories, cost data sources, price year, currency, and mean incremental costs), (7) perspective of economic evaluation, and (8) cost-effectiveness estimates, such as incremental costs (i.e., cost difference between IMI and comparator), incremental effects, ICER, and ICER acceptability for various WTP levels.

### Summary measures

Only base-cases analyses adhering to the intention-to-treat (ITT) principle were reported. Cost-effectiveness is ascertained when an intervention dominates the alternative, so it is both more effective and less costly or provides a greater outcome at higher costs that the society is willing to pay for^31^. In practice, interventions often show greater effects for higher costs. The efficacy of interventions is one of the indicators for their cost-effectiveness, as it represents the denominator of the ICER. Consequently, most often, the investment required for obtaining a favorable health outcome decreases with increasing effectiveness. Therefore, more effective treatments have a higher probability of being cost-effective. The relative effectiveness of an intervention is further influenced by its comparator, with smaller incremental effects in active comparator interventions to larger incremental effects in passive control groups^4^. Similarly, the level of therapist-led guidance in IMIs induces some effect moderation because it adds costs to an IMI, but may also enhance its effectiveness^4,83^. This is important when making conclusions about incremental cost-effectiveness. In this review, IMIs were judged to be cost-effective when:

- the IMI was dominant, i.e., the IMI’s effect was better, and its costs were lower than those of the comparator;
- the costs per QALY was below the WTP of £30,000 as suggested by the NICE^84^;
- studies using disease-specific clinical outcome such as treatment response, reliable change, were judged to be cost-effective when the probability of cost-effectiveness at a WTP of £0 was 80% or higher, which provides a high level of certainty for decision-making.

This means that the intervention is estimated to be more effective and costly in 80% of the cases. This criterion can be seen as conservative, as most interventions show higher effects at higher costs than alternative interventions. Again, as no thresholds for the WTP of these units of effect exist, applicable studies should be judged individually by decision-makers.

To facilitate comparison between countries, all national currencies were converted to Pound Sterling for the price year 2020^85^. First, the currency of the study was indexed to a 2020 equivalent by country-specific gross domestic product inflators (e.g., euro area 19) and then converted to Pound Sterling (£) using purchasing power parities^86^.

### Quality assessment

The quality of health-economic evaluations was assessed using the CHEC^79^. This 20-item checklist was developed to evaluate the methodological quality (internal and external validity) of economic evaluations. The total score is expressed as the percentage of the maximum score for each study. A summary quality score was calculated^24^ (percentage of criteria met by each study [range: 0–100%]) based on a scoring of “yes” (= 1), “suboptimal” (= 0.5), “no” (= 0), not applicable (NA)^24^. The following quality categories were used: excellent (100–95%), good (75–94%), average (50–74%), and poor (<50%).

In addition, Cochrane Collaboration’s tool for assessing RoB was used^87^ to determine selection, performance, detection, attrition, reporting, and other bias in research studies. Each item was rated as high, low, unclear RoB, or NA. Performance bias was not assessed, as participants and personnel cannot be blinded due to the nature of IMIs. Furthermore, detection bias was always rated as low, as IMIs commonly rely on self-report instruments. Incomplete outcome data were rated as low risk when data analysis was conducted in accordance with the ITT principle. RoB was converted to the Agency for Healthcare Research and Quality^88^ standards (i.e., good, fair, or poor quality). RoB and CHEC were rated independently by F.K. and C.B. Disagreement was discussed or resolved by a third reviewer (D.D.E.).

### Reporting summary

Further information on research design is available in the Nature Research Reporting Summary linked to this article.

## Supplementary information

Supplementary Information

Reporting Summary

## References

[1] Kessler RC (2009). The global burden of mental disorders: an update from the WHO World Mental Health (WMH) surveys. Epidemiol. Psichiatr. Soc..

[2] Steel Z (2014). The global prevalence of common mental disorders: a systematic review and meta-analysis 1980-2013. Int. J. Epidemiol..

[3] Walker ER, McGee RE, Druss BG (2015). Mortality in mental disorders and global disease burden implications a systematic review and meta-analysis. JAMA Psychiatry.

[4] Andrews G (2018). Computer therapy for the anxiety and depression disorders is effective, acceptable and practical health care: an updated meta-analysis. J. Anxiety Disord..

[5] Mack S (2015). Functional disability and quality of life decrements in mental disorders: results from the Mental Health Module of the German Health Interview and Examination Survey for Adults (DEGS1-MH). Eur. Psychiatry.

[6] Scott KM (2016). Association of mental disorders with subsequent chronic physical conditions. JAMA Psychiatry.

[7] Baglioni C (2016). Sleep and mental disorders: a meta-analysis of polysomnographic research. Psychol. Bull..

[8] GBD 2015 DALYs and HALE Collaborators. (2016). Global, Regional, and National Disability-adjusted Life Years (Dalys) for 315 diseases and injuries and healthy life expectancy (Hale), 1990-2015: a systematic. Lancet.

[9] Vigo D, Thornicroft G, Atun R (2016). Estimating the true global burden of mental illness. Lancet Psychiatry.

[10] Bloom, D. E. et al. The global economic burden of non-communicable diseases. http://www3.weforum.org/docs/WEF_Harvard_HE_GlobalEconomicBurdenNonCommunicableDiseases_2011.pdf (2011).

[11] Gustavsson A (2011). Cost of disorders of the brain in Europe 2010. Eur. Neuropsychopharmacol..

[12] Cuijpers P (2013). The efficacy of psychotherapy and pharmacotherapy in treating depressive and anxiety disorders: a meta-analysis of direct comparisons. World Psychiatry.

[13] Kazdin AE (2017). Addressing the treatment gap: a key challenge for extending evidence-based psychosocial interventions. Behav. Res. Ther..

[14] De Girolamo G, Dagani J, Purcell R, Cocchi A, McGorry PD (2012). Age of onset of mental disorders and use of mental health services: needs, opportunities and obstacles. Epidemiol. Psychiatr. Sci..

[15] Andrade LH (2014). Barriers to mental health treatment: results from the WHO World Mental Health surveys. Psychol. Med..

[16] Griffiths F, Lindenmeyer A, Powell J, Lowe P, Thorogood M (2006). Why are health care interventions delivered over the internet? A systematic review of the published literature. J. Med. Internet Res..

[17] Ebert DD, Cuijpers P, Muñoz RF, Baumeister H (2017). Prevention of mental health disorders using internet- and mobile-based interventions: a narrative review and recommendations for future research. Front. Psychiatry.

[18] Sander L (2016). Effectiveness of internet-based interventions for the prevention of mental disorders: a systematic review and meta-analysis. JMIR Ment. Heal.

[19] Rosenbaum S, Newby JM, Steel Z, Andrews G, Ward PB (2015). Online physical activity interventions for mental disorders: a systematic review. Internet Inter..

[20] Stratton E (2017). Effectiveness of eHealth interventions for reducing mental health conditions in employees: a systematic review and meta-analysis. PLoS ONE.

[21] Gomes M, Murray E, Raftery J (2022). Economic evaluation of digital health interventions: methodological issues and recommendations for practice. Pharmacoeconomics.

[22] Hedman E, Ljótsson B, Lindefors N (2012). Cognitive behavior therapy via the internet: a systematic review of applications, clinical efficacy and cost-effectiveness. Expert Rev. Pharmacoecon. Outcomes Res..

[23] Arnberg FK, Linton SJ, Hultcrantz M, Heintz E, Jonsson U (2014). Internet-delivered psychological treatments for mood and anxiety disorders: a systematic review of their efficacy, safety, and cost-effectiveness. PLoS ONE.

[24] Ophuis RRH (2017). Cost-effectiveness of interventions for treating anxiety disorders: a systematic review. J. Affect. Disord..

[25] Paganini S, Teigelkötter W, Buntrock C, Baumeister H (2018). Economic evaluations of internet- and mobile-based interventions for the treatment and prevention of depression: a systematic review. J. Affect. Disord..

[26] Conejo-Cerón S (2021). Health-economic evaluation of psychological interventions for depression prevention: systematic review. Clin. Psychol. Rev..

[27] Kolovos S (2018). Cost effectiveness of guided Internet-based interventions for depression in comparison with control conditions: an individual–participant data meta-analysis. Depress Anxiety.

[28] Donker T (2015). Economic evaluations of internet interventions for mental health: a systematic review. Psychol. Med..

[29] Mitchell, L., Joshi, U., Patel, V., Lu, C. & Naslund, J. A. Economic evaluations of internet-based psychological interventions for anxiety disorders and depression: a systematic review. *J. Affect. Disord*. **284**, 157–182 (2021). 10.1016/j.jad.2021.01.092 PMC8008508 33601245

[30] Karyotaki E, Tordrup D, Buntrock C, Bertollini R, Cuijpers P (2017). Economic evidence for the clinical management of major depressive disorder: a systematic review and quality appraisal of economic evaluations alongside randomised controlled trials. Epidemiol. Psychiatr. Sci..

[31] Drummond, M., Sculpher, M. J., Claxton, K., Stoddart, G. L. & Torrance, G. W. *Methods for the Economic Evaluation of Health Care Programmes* (Oxford University Press, 2015).

[32] Ebert DD (2018). A health economic outcome evaluation of an internet-based mobile-supported stress management intervention for employees. Scand. J. Work. Environ. Health.

[33] Kählke F (2019). Economic evaluation of an internet-based stress management intervention alongside a randomized controlled trial. JMIR Ment. Heal..

[34] Buntrock C (2021). Guided internet-based cognitive behavioral therapy for insomnia: health-economic evaluation from the societal and public health care perspective alongside a randomized controlled trial. J. Med. Internet Res..

[35] Thiart H (2016). Internet-based cognitive behavioral therapy for insomnia: a health economic evaluation. Sleep.

[36] Hedman E (2011). Cost-effectiveness of internet-based cognitive behavior therapy vs. cognitive behavioral group therapy for social anxiety disorder: results from a randomized controlled trial. Behav. Res. Ther..

[37] Hedman E (2014). Clinical effectiveness and cost-effectiveness of internet- vs. group-based cognitive behavior therapy for social anxiety disorder: 4-year follow-up of a randomized trial. Behav. Res. Ther..

[38] El Alaoui S, Hedman-Lagerlof E, Ljotsson B, Lindefors N (2017). Does internet-based cognitive behaviour therapy reduce healthcare costs and resource use in treatment of social anxiety disorder? A cost-minimisation analysis conducted alongside a randomised controlled trial. BMJ Open.

[39] Brabyn S (2016). The second Randomised Evaluation of the Effectiveness, cost-effectiveness and Acceptability of Computerised Therapy (REEACT-2) trial: does the provision of telephone support enhance the effectiveness of computer-delivered cognitive behaviour therapy? A randomised controlled trial. Health Technol. Assess..

[40] Littlewood E (2015). A randomised controlled trial of computerised cognitive behaviour therapy for the treatment of depression in primary care: the Randomised Evaluation of the Effectiveness and Acceptability of Computerised Therapy (REEACT) trial. Health Technol. Assess..

[41] Phillips R (2014). Randomized controlled trial of computerized cognitive behavioural therapy for depressive symptoms: effectiveness and costs of a workplace intervention. Psychol. Med..

[42] Rantz M (2017). Randomized trial of intelligent sensor system for early illness alerts in senior housing. J. Am. Med. Dir. Assoc..

[43] Buntrock C (2017). Preventing depression in adults with subthreshold depression: health-economic evaluation alongside a pragmatic randomized controlled trial of a web-based intervention corresponding author. J. Med. Internet Res..

[44] Klein NS (2018). Economic evaluation of an internet-based preventive cognitive therapy with minimal therapist support for recurrent depression: randomized controlled trial. J. Med. Internet Res..

[45] Bergstrom J (2010). Internet-versus group-administered cognitive behaviour therapy for panic disorder in a psychiatric setting: a randomised trial. BMC Psychiatry.

[46] Nordgren LB (2014). Effectiveness and cost-effectiveness of individually tailored Internet-delivered cognitive behavior therapy for anxiety disorders in a primary care population: a randomized controlled trial. Behav. Res Ther..

[47] Landis JR, Koch GG (1977). The measurement of observer agreement for categorical data. Biometrics.

[48] Bolier L (2014). Cost-effectiveness of online positive psychology: randomized controlled trial. J. Posit. Psychol..

[49] Yan C, Rittenbach K, Souri S, Silverstone PH (2019). Cost-effectiveness analysis of a randomized study of depression treatment options in primary care suggests stepped-care treatment may have economic benefits. BMC Psychiatry.

[50] Gerhards SAH (2010). Economic evaluation of online computerised cognitive-behavioural therapy without support for depression in primary care: randomised trial. Br. J. psychiatry.

[51] Nobis S (2018). Web-based intervention for depressive symptoms in adults with types 1 and 2 diabetes mellitus: a health economic evaluation. Br. J. Psychiatry.

[52] Geraedts AS (2015). Economic evaluation of a web-based guided self-help intervention for employees with depressive symptoms: results of a randomized controlled trial. J. Occup. Environ. Med..

[53] Warmerdam L, Smit F, van Straten A, Riper H, Cuijpers P (2010). Cost-utility and cost-effectiveness of Internet-based treatment for adults with depressive symptoms: randomized trial. J. Med. Internet Res..

[54] Hollinghurst S (2010). Cost-effectiveness of therapist-delivered online cognitive-behavioural therapy for depression: randomised controlled trial. Br. J. Psychiatry.

[55] van Luenen S, Kraaij V, Garnefski N, Spinhoven P, van den Akker-van Marle ME (2019). Cost-utility of a guided Internet-based intervention in comparison with attention only for people with HIV and depressive symptoms: a randomized controlled trial. J. Psychosom. Res..

[56] Titov N (2015). Clinical and cost-effectiveness of therapist-guided internet-delivered cognitive behavior therapy for older adults with symptoms of anxiety: a randomized controlled trial (Provisional abstract). Behav. Ther..

[57] Romero-Sanchiz P (2017). Economic evaluation of a guided and unguided internet-based CBT intervention for major depression: results from a multicenter, three-armed randomized controlled trial conducted in primary care. PLoS ONE.

[58] Hedman E (2013). Cost-effectiveness and long-term effectiveness of internet-based cognitive behaviour therapy for severe health anxiety. Psychol. Med..

[59] Dear BF (2015). Clinical and cost-effectiveness of therapist-guided internet-delivered cognitive behavior therapy for older adults with symptoms of anxiety: a randomized controlled trial (Provisional abstract). Behav. Ther..

[60] Hedman E, Andersson E, Ljotsson B, Axelsson E, Lekander M (2016). Cost effectiveness of internet-based cognitive behaviour therapy and behavioural stress management for severe health anxiety. BMJ Open.

[61] Powell J (2020). Effectiveness and cost-effectiveness of a self-guided internet intervention for social anxiety symptoms in a general population sample: randomized controlled trial. J. Med. Internet Res..

[62] Rohr, S. et al. A self-help app for Syrian refugees with posttraumatic stress (Sanadak): randomized controlled trial. *JMIR mHealth uHealth* **9**, e24807 (2021). 10.2196/24807 PMC7935251 33439140

[63] Andersson E (2015). Cost-effectiveness of internet-based cognitive behavior therapy for obsessive-compulsive disorder: results from a randomized controlled trial. J. Obsessive. Compuls. Relat. Disord..

[64] Andersson E (2015). Cost-effectiveness of an internet-based booster program for patients with obsessive-compulsive disorder: results from a randomized controlled trial (Provisional abstract). J. Obsessive. Compuls. Relat. Disord..

[65] Lenhard F (2017). Cost-effectiveness of therapist-guided internet-delivered cognitive behaviour therapy for paediatric obsessive-compulsive disorder: results from a randomised controlled trial. BMJ Open.

[66] Lovell K (2017). Clinical effectiveness, cost-effectiveness and acceptability of low-intensity interventions in the management of obsessive-compulsive disorder: the Obsessive-Compulsive Treatment Efficacy randomised controlled Trial (OCTET). Health Technol. Assess..

[67] De Bruin EJ (2016). Cost-effectiveness of group and internet cognitive behavioral therapy for insomnia in adolescents: results from a randomized controlled trial. Sleep.

[68] Lindsäter E (2019). Cost-effectiveness of therapist-guided internet-based cognitive behavioral therapy for stress-related disorders: secondary analysis of a randomized controlled trial. J. Med. Internet Res..

[69] Ebert, D. D. et al. A health economic outcome evaluation of an internet-based mobile-supported stress management intervention for employees. *Scand. J. Work. Environ. Heal*. **44**, 171–182 (2018). 10.5271/sjweh.3691 29144535

[70] van Spijker BAJ (2012). Reducing suicidal ideation: cost-effectiveness analysis of a randomized controlled trial of unguided web-based self-help. J. Med. Internet Res..

[71] McDougall JA, Furnback WE, Wang BCM, Mahlich J (2020). Understanding the global measurement of willingness to pay in health. J. Mark. Access Heal. Policy.

[72] Shiroiwa T (2010). International survey on willingness-to-pay (WTP) for one additional QALY gained: what is the threshold of cost effectiveness?. Health Econ..

[73] Kan K (2021). More than cost-effectiveness? Applying a second-stage filter to improve policy decision making. Heal. Expect..

[74] Fenwick E, O’Brien B, Briggs A (2004). Cost-effectiveness acceptability curves: facts, fallacies and frequently asked questions. Health Econ..

[75] Kendrick T (2022). Management of depression in adults: summary of updated NICE guidance. BMJ.

[76] Gerlinger G, Mangiapane N, Sander J (2021). Digital health applications (DiGA) in medical and psychotherapeutic care. Opportunities and challenges from the perspective of the healthcare providers. Bundesgesundheitsblatt Gesundheitsforsch Gesundheitsschutz.

[77] Ramsey SD (2015). Cost-effectiveness analysis alongside clinical trials II—an ISPOR Good Research Practices Task Force Report. Value Heal.

[78] Husereau D (2013). Consolidated Health Economic Evaluation Reporting Standards (CHEERS) statement. Eur. J. Heal. Econ..

[79] Thielen, F. W. et al. How to prepare a systematic review of economic evaluations for clinical practice guidelines: database selection and search strategy development (part 2/3). *Expert Rev. Pharmacoecon. Outcomes Res.* **16**, 705–721 (2016). 10.1080/14737167.2016.1246962 27805466

[80] Page MJ (2021). The PRISMA 2020 statement: an updated guideline for reporting systematic reviews. Syst. Rev..

[81] van Mastrigt GAPG (2016). How to prepare a systematic review of economic evaluations for informing evidence-based healthcare decisions: a five-step approach (part 1/3). Expert Rev. Pharmacoecon. Outcomes Res.

[82] PROSPERO Registry. https://www.crd.york.ac.uk/PROSPERO/display_record.php?RecordID=93808 (2018).

[83] Karyotaki E (2018). Is self-guided internet-based cognitive behavioural therapy (iCBT) harmful? An individual participant data meta-analysis. Psychol. Med..

[84] McCabe C, Claxton K, Culyer AJ (2008). The NICE cost-effectiveness threshold: what it is and what that means. Pharmacoeconomics.

[85] Organisation for Economic Co-operation and Development (OECD). PPP and exchanges rates. https://data.oecd.org/conversion/purchasing.power-parties-ppp.htm (2018).

[86] Tate DF, Finkelstein EA, Khavjou O, Gustafson A (2009). Cost effectiveness of internet interventions: review and recommendations. Ann. Behav. Med..

[87] Higgins, J. P. T. & Altman, D. G. Assessing risk of bias in included studies. In *Cochrane Handbook for Systematic Reviews of Interventions: Cochrane Book Series (*eds. Higgins, J. P. T. & Green, S.) 187–241 (WILEY, 2008). 10.1002/9780470712184.ch8.

[88] Viswanathan M (2018). Recommendations for assessing the risk of bias in systematic reviews of health-care interventions. J. Clin. Epidemiol..

