# Supplementary material for: Systematic review of economic evaluations for internet- and mobile-based interventions for mental health problems
Source: NPJ Digit Med. 2022 Nov 23;5:175. doi: 10.1038/s41746-022-00702-w (PMC9686241; doi:10.1038/s41746-022-00702-w)
Supplement: Supplementary file 1 — Supplementary Information [file 41746_2022_702_MOESM1_ESM.pdf]

## Supplementary table 1: Health economic outcomes

| Author<br><br>Perspective (currency year) | Cost categories | Δ Costs | Health-related outcome | ICER[a] | CEA[b]<br><br>P (WTP = 0); WTP (*p* = 0.5); WTP (*p* = 0.95) | QALY | ICUR[a] | CUA[b]<br><br>P (WTP = 0; £20,000; £30,000) | CBA | Results[c] |
|---|---|---|---|---|---|---|---|---|---|---|
| Boiler, 2014<br><br>Societal (2009, €) | - Direct costs: medical, non-medical, intervention<br>- Indirect costs: productivity losses<br><br>Source: TiC-P | €1,471 | Symptom severity (CES-D < 16, 5 score change)**[d] | €9,807 NE | 22%; >€100,000 | N.A. | N.A. | N.A. | N.A. | Unguided iPPI vs. WLC:<br><br>ICER: £11,226 per symptom severity<br><br>iPPI generated higher effects at higher costs. |
| Buntrock, 2017<br><br>Societal, Healthcare (2013, €) | - Direct costs: healthcare, patient costs (e.g., traveling, opportunity), medication, intervention<br>- Indirect costs: productivity losses<br><br>Source: TiC-P | Societal: €143<br><br>Healthcare: €136 | Depression free years (DFY)**[e] | Societal: €1,117 NE<br><br>Healthcare: €1,125 NE | Societal: 38%; €2,000[f] €9,680<br><br>Healthcare: 17%; €3,920[f] | EQ-5D-3L | Societal: €13,400 NE<br><br>Healthcare: €13,500 NE | Societal: 38%[f], ≥60%[f]<br><br>Healthcare: N.R., ≥ 64% | N.A. | Guided iCBT vs. TAU[+]:<br><br>ICER: £1,170 (societal), £1,178 (healthcare)/depression-free year<br>ICUR: £14,034 (societal), £14,139 (healthcare)/QALY gained<br>iPST/BA resulted in greater effects and more QALY gained at higher costs. |
| Gerhards, 2010<br><br>Societal (2007, €) | - Direct costs: medical costs (healthcare sector), non-medical costs (travel, use of iCBT) intervention<br>- Indirect costs: productivity losses<br><br>Source: PRODISQ, patient reported healthcare use | iCBT vs. TAU: €-711<br><br>iCBT+TAU vs. TAU: €738<br><br>iCBT vs. iCBT+TAU: €-1,449 | Symptom severity (BDI-II): reliable change index | N.R. | iCBT:70[f]<br><br>TAU:13[f]<br>iCBT + TAU[+]: 18[f], outper-forms others with increased WTP (max. 80%) | EQ-5D | N.A. | iCBT:65%[f], 57%[f], 55%[f]<br><br>TAU: 25%[f], 30%[f], 33%[f]<br>iCBT & TAU: 10%[f], 12%[f], 14%[f] | N.A. | Unguided iCBT vs. unguided iCBT + TAU[+] vs. TAU[Nb]<br><br>Costs were lowest for the iCBT. There were no significant group differences in effect or QALY compared to iCBT plus TAU+ and TAU only. CUA and CEA tend to be in favor of iCBT. |
| Phillips, 2014<br><br>N.R. (N.R.., £) | - Direct costs: hospital, community, and healthcare services<br>- Indirect costs: lost workdays (total absence)<br><br>Source: CSSRI, self-assessed absence from work | £-35 | N.A. | N.A. | N.A. | EQ-5D | N.R., SW | N.R. | N.A. | Unguided iCBT vs. AC:<br><br>iCBT is less costly but generates lower QALYs. |

| Author | Cost categories | Δ Costs | Outcome | ICER | CEA | QALY | ICUR | CUA | CBA | Results |
|---|---|---|---|---|---|---|---|---|---|---|
| Titov, 2015<br><br>Healthcare, (N.R., AU$) | - Direct costs: healthcare resource use (admissions, consultations, medication use), intervention (therapist´s time)<br><br>Source: resource use, health-care visits | $52 | N.A. | N.A. | N.A. | EQ-5D-5L*[d] | AU$4,392 NE | 8%[e], 95%[e], 95%[e] | N.A. | Guided iCBT vs. WLC:<br><br>ICUR: £2,466/QALY gained<br><br>iCBT resulted more QALY at higher costs. |
| Van Luenen, 2019<br><br>Societal, Healthcare (2017, €) | - Direct costs: intervention, healthcare<br>- Indirect costs: productivity losses<br><br>Source: TiC-P | Societal: €-731<br><br>Healthcare: €11 | N.A. | N.A. | N.A. | SF-6D | Societal: N.R., SE<br><br>Healthcare: N.R., NE | Societal: 95%[f],96%[f], 98%[f]<br>Healthcare: 48%[f], 88%[f], 94%[f] | N.A. | Guided iCBT vs. AC + WLC:<br><br>iCBT generated less (societal) and higher (healthcare) costs/QALY gained. |
| Brabyn, 2016<br><br>Healthcare (2012/13, £) | - Direct costs: service use, medication, intervention (only telephone support)<br><br>Source: CSSRI | £591 | N.A. | N.A. | N.A. | EQ-5D-5L | N.R., SE | 50%, 55%, 55% | N.A. | Guided iCBT vs. unguided iCBT:<br><br>Guided iCBT resulted in more QALYs gained at lower costs. |
| Geraedts 2015<br><br>Societal, Employer (2012, €) | - Direct costs: medical/non-medical costs, occupational health, intervention<br>- Indirect costs: productivity losses<br><br>Source: TiC-P, WHO-HPQ | Societal: €-714<br><br>Employer: €-508 | CES-D, clinically significant change | Societal: €-6,645 SE<br><br>Employer: €-4,664 SE | Societal: 62%, N.A., €44,000<br><br>Employer: 55%, N.A., €115,000 | EQ-5D-3 L | Societal: €532,959 SW<br><br>Employer: €382,354 SW | Societal: N.R.; max. 62%<br><br>Employer: N.R.; max. 55% | NB: €508<br><br>BCR: €2.8 | Guided iPST vs. TAU[Nb]:<br><br>ICER: £-6,486 (societal), £-4,552 (employer)/significant change<br>ICUR: £520,203 (societal), £373,203 (employer)/QALY gained<br><br>iPST dominated regarding clinically significant change but resulted in less QALYs gained at lower costs. |
| Hollinghurst, 2010<br><br>Healthcare (inferred societal) (2007, £) | - Direct costs: NHS service use, personal expenditures, intervention (no costs for IMI/website)<br>- Indirect costs: productivity (workdays lost)<br><br>Source: resource use diary | £469 | BDI "Recovery" (BDI <10) | £3,528 NE | N.R. | EQ-5D | £17,173 NE | 0%[f]; 56%[f], 75%[f] | N.A. | Guided iCBT vs. WLC[Nb]:<br><br>ICER: £4,686/recovered person<br>ICUR: £22,810/ QALY gained<br><br>iCBT resulted in greater effects and more QALY gained at higher costs. |

| Author | Cost categories | Δ Costs | Outcome | ICER | CEA | QALY | ICUR | CUA | CBA | Results |
|---|---|---|---|---|---|---|---|---|---|---|
| Klein, 2018<br><br>Societal, (2014, €) | - Direct costs: intervention, healthcare, participant<br>- Indirect costs: productivity losses<br><br>Source: TiC-P | €1008 | Depression-free days | N.R., NE | 40%, €50[f], max 65% | EQ-5D-3L | N.R., NE | 18%, max 40% | N.A. | iPCT +TAU vs. TAU:<br><br>iPCT resulted in greater effects and more QALY gained at higher cost. |
| Littlewood, 2015<br><br>Healthcare (2011/12, £) | - Direct costs: primary care, hospital, other community services, medication, intervention<br><br>Source: GP medical records | iCBT1 vs. uGPC: £104<br><br>iCBT2 vs. uGPC: £-106 | N.A. | N.A. | N.A. | EQ-5D | iCBT1 vs. TAU: £6,933 SW<br><br>iCBT2 vs. TAU: dominated NW | iCBT1: 94%[f]; 42%[f] 38%[f]<br><br>iCBT2: 0%[f], 0.04%[f]; 4%[f]<br><br>uGPC: 6%[f]; 55%, 58%[f] | N.A. | Unguided iCBT 1 vs. uGPC [Nb]:<br><br>ICUR: £7,838/QALY gained iCBT1 resulted in lower costs and less QALY gained.<br><br>Unguided iCBT 2 vs. uGPC:<br><br>iCBT2 resulted in higher costs and less QALY gained. uGPC alone compared with iCBT was most likely to be cost-effective. |
| Nobis, 2018<br><br>Societal (2013, €) | - Direct costs: healthcare services, medication, travel expenses, intervention<br>- Indirect costs: domestic help, productivity losses<br><br>Source: TiC-P | €97 | Treatment response (CES-D): Reliable change Index and ≤ 23 | €233 NE | 48%; €250[f] €4,800[f] | EQ-5D-3L | €10,708 NE | 46%; N.R., €14,000 = 51% | N.A. | Guided iCBT (1) vs. TAU+:<br><br>ICER: £244/treatment response<br>ICUR: £11,215/QALY gained<br><br>iCBT resulted in greater effects and more QALY gained at higher cost |
| Romero-Sanchiz, 2017<br><br>Societal (2014, €) | - Direct costs: healthcare sector (GP, hospital stays), medication<br>-Indirect costs: absenteeism<br><br>Source: CSSRI | iCBT1 vs. TAU$^+$: €-409<br><br>iCBT2 vs. TAU$^+$: €41 | BDI-II, point improvement*[g] | iCBT1 €-98 SE<br><br>iCBT2 €10 NE | N.R. | EQ-5D-3 L*[g] | iCBT1: €-5,160 SE<br><br>iCBT2: €497 NE | N.R. | N.A. | Unguided iCBT1 vs. TAU$^+$:<br><br>ICER: £-99/point improvement<br>ICUR: £-5,201 per QALY<br>iCBT1 dominated TAU$^+$<br><br>Guided iCBT2 vs. TAU$^+$:<br><br>ICER: £10/point improvement<br>ICUR: £501/QALY gained<br>iCBT2 resulted in greater effects and more QALY gained at higher cost. |

| Author | Cost categories | Δ Costs | Outcome | ICER | CEA | QALY | ICUR | CUA | CBA | Results |
|---|---|---|---|---|---|---|---|---|---|---|
| Warmerdam, 2010<br><br>Societal (2007, €) | - Direct costs: medical/non-medical costs (e.g., traveling, parking), intervention<br>-Indirect costs: domestic help, productivity losses<br><br>Source: TiC-P | iCBT vs. WLC: €256<br><br>iPST vs. WLC: €147<br><br>iCBT vs. iPST: €109 | Clinically significant change (CES-D < 16) | iCBT vs. WLC: €1,817 NE<br><br>iPST vs. WLC: €1,248 NE<br><br>iCBT vs. iPST: €-36, NW | iCBT vs. WLC: 30%, €2,500[f], €25.000[f]<br><br>iPST vs. WLC: 38%, €2,000[f], €35.000[f]<br><br>iCBT vs. iPST N.R. | EQ-5D | iCBT vs. WLC: €22,609 NE<br><br>iPST vs. WLC: €11,523 NE<br><br>iCBT vs. iPST: N.R. | iCBT vs. WLC: 28%; 44%[f], 55%[f]<br><br>iPST vs. WLC: 38%; 52%[f], 63%[f]<br><br>iCBT vs. iPST: N.R. | N.A. | Guided iCBT vs. WLC:<br>ICER: £2,147/clinical change<br>ICUR: £26,713/QALY gained<br><br>Guided iPST vs. WLC:<br>ICER: £1,475/clinical change<br>ICUR: £13,615/QALY gained<br><br>iCBT vs. iPST:<br>ICER: £43/clinical change<br><br>iCBT and iPST resulted in greater effects and more QALY gained at higher costs compared to WLC. |
| Yan, 2019<br><br>Healthcare (2017, $) | -Direct costs: healthcare (physician, outpatient, inpatient services e.g., salaries, drugs, medical supplies)<br><br>Source: Health administrative databases | SCP vs.SC: -$155<br><br>SCP vs. TAU: -$449<br><br>SCP vs. iCBT: -$161 | N.A. | N.A. | N.A. | EQ-5D-5L | N.R. | SC: 26%[f], 27%[f], 28%[f]<br><br>TAU: 18%[f], 11%[f], 7%[f]<br><br>iCBT: 27%[f], 29%[f],30%[f]<br><br>SCP: 29%[f], 33%[f], 34%[f] | N.A. | SCP vs. SC, TAU, unguided iCBT[Nb]:<br><br>QALYs were highest in SCP, followed by SC, CBT, and TAU.<br><br>SCP had a higher probability for cost-effectiveness than the three alternatives. |
| Bergström, 2010<br><br>N. R., (N.R., €) | -Direct cost: therapist´s and psychiatrist´s time<br><br>Source: self-assessed therapist time | €-239 | Responder ≥ 40% reduction on PDSS | N.R. SE | 75%; N.R. | N.A. | N.A. | N.A. | N.A. | Guided iCBT vs. gCBT:<br><br>iCBT dominates gCBT, generating more effect (responder) at less costs. |
| Dear, 2015<br><br>Healthcare (2013, AU$) | -Direct costs: primary, secondary care, therapist or supervisor costs, medication, internet access, computer, telephone use<br><br>Source: patients’ healthcare resource use | $92 | N.A. | N.A. | N.A. | EQ-5D-5L*[h] | $8,806 NE | 0%[f], 93%[f], 96%[f] | N.A. | Guided iCBT vs. WLC:<br><br>ICUR: £4,825/QALY gained<br><br>iCBT resulted in more QALYs at higher costs. |

| Author | Cost categories | Δ Costs | Outcome | ICER | CEA | QALY | ICUR | CUA | CBA | Results |
|---|---|---|---|---|---|---|---|---|---|---|
| Nordgren, 2014<br><br>N.R.<br>(N.R., US$) | - Direct cost: healthcare, participant medication, intervention (therapists)<br>- Indirect costs: productivity losses<br><br>Source: TiC-P | CEA $-616<br>CUA $-474 | CORE-OM defined as responder **[h] | $-1,824 SE | 95%; N.A. | EQ-5D, N.R. | $-7,523, SE | 90%, > 95% | N.A. | Guided iCBT vs. AC:<br><br>ICERs: £-1,254/responder<br>ICUR: £-5,173/QALY gained<br><br>iCBT generated less costs and more effects and QALY gained. |
| Hedman, 2013<br><br>Societal, (2010, £), | -Direct costs: intervention (only therapists), healthcare, participant<br>-Indirect costs: productivity losses<br><br>Source: TiC-P | £-784 | HAI, no diagnostic criteria for severe health anxiety**[i] | £-1,244 SE | 64%; £4.800[f] | EQ-5D**[i] | £−6,533 SE | 67%, 91%[f] | N.A. | Guided iCBT vs AC:<br><br>ICER £-1,512/person without diagnosis on HAI<br>ICUR; £-7,940/QALY gained<br><br>iCBT generated less costs per effect and QALY gained. |
| Hedman, 2016<br><br>Societal, (2013, US$) | -Direct cost: intervention (only therapists), healthcare, participant costs<br>- Indirect costs: productivity losses<br><br>Source: TiC-P | $310 | HAI, responder clinically significant improvement*[i] | $2,214 NE | 9%; $2,300; $20,000[f] | EQ-5D, N.R. | $10,000 NE | N.R. | N.A. | Unguided iCBT vs. unguided iMA[Nb]:<br><br>ICER: £1,633/responder<br>ICUR £7,376/QALY gained<br><br>iCBT resulted in higher effect and more QALYs gained at higher costs. |
| Hedman, 2011<br><br>Societal, (2009, US$) | -Direct costs: intervention (only therapists), healthcare, participant costs<br>-Indirect costs: productivity losses<br><br>Source: TiC-P | $-1335 | LSAS < 43.3, responder | $-7,046 SE | 81%; $70,000[f] | EQ-5D | $-17, 823 SE | 81%, 82%[f],79%[f] | N.A. | Guided iCBT vs. gCBT:<br><br>ICER: £-5,729/responder<br>ICUR £-14,491/QALY gained<br><br>iCBT generated more effect and QALY gained at less costs. |
| Hedman, 2014<br><br>Societal, (N.R., US$) | - Direct costs: intervention (only therapists), healthcare, participant costs<br>- Indirect costs: productivity losses<br><br>Source: TiC-P | $-808 | LSAS < 43.3, responder | $10,100 SW | 62%, max. 62% | EQ-5D | -$7,345 SE | Max. 62%, 39%[f], 34%[f] | N.A. | Guided iCBT vs. gCBT:<br><br>ICER: £11,749/responder iCBT resulted in less effect at lower costs.<br><br>ICUR: £-8,544/QALY gained iCBT generated less costs per QALY gained. |

| **Author** | **Cost categories** | **Δ Costs** | **Outcome** | **ICER** | **CEA** | **QALY** | **ICUR** | **CUA** | **CBA** | **Results** |
|---|---|---|---|---|---|---|---|---|---|---|
| Alaoui, 2017<br><br>Healthcare (2017, €) | - Direct costs: hospital space, IT usage, security, management, therapists<br><br>Source: TDABC | €-343 | N.A. | N.A. | N.A. | EQ-5D | N.R. | 100%[f], 68%[f], 67%[f] | N.A. | Guided iCBT vs. gCBT:<br><br>Both treatments were equally efficacious but iCBT generated less costs. |
| Powell, 2020<br><br>Societal, Healthcare, (2016/17, £) | - Direct costs: healthcare costs, intervention<br>- Indirect costs: workdays lost<br><br>Source: PSSRU, UK | Social: £-65<br>Healthcare: £-63 | N.A. | N.A. | N.A. | SF-6D | Social: N.R SE<br><br>Healthcare: N.R SE | N.R. | N.A. | Unguided iCBT vs. WLC:<br><br>iCBT resulted in less costs per QALY gained. |
| Andersson, 2015a<br><br>Societal Healthcare (2013, US$) | - Direct costs: therapist, healthcare, participant costs<br>- Indirect costs: productivity losses<br><br>Source: TiC-P | Societal $503<br><br>Healthcare $336 | Y-BOCS, additional remission**[j] | Societal $931 NE<br><br>Healthcare $672 NE | Societal 15%[f], $ 1100, $2600 = 90%<br><br>Healthcare 0%[f], $700[f], $900[f] | EQ-5D-5L**[k] | Societal $7,186 NE<br><br>Healthcare $4,800 NE | Societal 15%[f]; >95%[f]<br><br>Healthcare 0%[f]; >90%[f] | N.A. | Guided iCBT vs. AC[Nb]:<br><br>ICER: £687 (societal), £496 (healthcare)/remission<br>ICUR: £5,301 (societal), £3,541 (healthcare)/QALY gained<br><br>iCBT resulted in greater effects and more QALYs at higher costs. |
| Andersson, 2015b<br><br>Societal, Healthcare (2013, US$) | - Direct costs: therapists, healthcare, participant<br>- Indirect costs: productivity losses<br><br>Source: TiC-P | Societal: $338<br><br>Healthcare: $242 | Y-BOCS, relapse avoided | Societal: $1,489 NE<br><br>Healthcare: $1,066 NE | Societal: 18%[f], $1500[f], $7000[e]<br><br>Healthcare 0%, $900[f], $4250[f] | N.A. | N.A. | N.A. | N.A. | iCBT booster session vs. TAU[Nb]:<br><br>ICER: £1,098 (societal), £786 (healthcare)/relapse avoided<br><br>The additional booster session generated higher effects at higher costs. |
| Lenhard, 2016<br><br>Societal, Healthcare (2016, US$) | - Direct costs: healthcare use, supportive resources, drugs, intervention<br>- Indirect costs: school absence, productivity losses<br><br>Source: TiC-P | Societal: $-145<br><br>Healthcare: $21 | Treatment responder >34% on CY-BOCS **[l] | Societal: N.R., SE<br><br>Healthcare: $78 NE | Societal: 59.4%; N.R.<br><br>Healthcare: 48%[f]; $200[f], $3000[f] | EQ-5D-Y | Societal: N.R. SW<br><br>Healthcare: N.R. | N.R. | N.A. | Guided iCBT vs. WLC[Nb]:<br><br>Societal:<br>iCBT generated less costs per treatment responder.<br>iCBT generated slightly less QALY at lower cost.<br><br>Healthcare:<br>ICER: £57/ treatment responder<br>iCBT produced higher effects at higher costs. |

| Author | Cost categories | Δ Costs | Outcome | ICER | CEA | QALY | ICUR | CUA | CBA | Results |
|---|---|---|---|---|---|---|---|---|---|---|
| Lovell, 2017<br><br>Societal, Healthcare (2013/14, £) | - Direct costs: hospital and community-based health- and social-care services, medication, out-of-pocket expenses and savings, intervention<br>- Indirect costs: productivity losses<br><br>Source: AD-SUS, WHO´s HPQ | Healthcare<br>iCBT vs. WLC: £138<br>SH vs. WLC: £364<br>iCBT vs. SH: £-226<br><br>Societal<br>iCBT vs. WLC: £200<br>SH vs. WLC: £310<br>iCBT vs. SH: £-109 | N.A. | N.A. | N.A. | EQ-5D-3L QALY | Healthcare<br>iCBT vs. WLC: £32,857 NE<br><br>SH vs. WLC: £55,152 NE<br><br>iCBT vs. SH: £94,167 SW<br><br>Societal<br>iCBT vs.WLC: £48,095 NE<br>SH vs. WLC: £46,970 NE<br>iCBT vs. SH: £45,417 SW | Healthcare<br>iCBT vs. WLC:<br>17%[f], 42%[e], 52%[f]<br>SH vs. WLC:<br>0%[e], 0%[f], 8%[f]<br>iCBT vs. SH:<br>95%[f], 88%[f], 85%[f]<br><br>Societal<br>iCBT vs. WLC:<br>15%[f], 28%[f], 35%[f]<br>SH vs. WLC:<br>0%[f], 5%[f], 15%[f]<br>iCBT vs. SH:<br>72%[f], 65%[f], 60%[f] | N.A. | Guided iCBT & Self-help vs. WLC[Nb]:<br><br>Healthcare:<br>ICUR: iCBT (£35,699), SH (£59,923)/QALY gained<br><br>Societal:<br>ICUR: iCBT (£52,255), SH (£51,033)/QALY gained<br><br>iCBT and SH gained more QALY at higher costs.<br><br>iCBT vs. Self-help:<br><br>ICUR: £102,312 (healthcare), £49,346 (societal)/QALY gained<br><br>iCBT had less costs and slightly less QALY gained. |
| Röhr, 2021<br><br>Healthcare (2019, €) | - Direct costs: healthcare costs, intervention,<br><br>Source: CSSRI | €-100 | N.A. | N.A. | N.A. | EQ-5D-5L | N.R. | 81%; 38% 27% | N.A. | Unguided iCBT vs. TAU[+Nb]:<br><br>iCBT generated lower costs per QALY gained. |
| De Bruin, 2016<br><br>Societal (2014, €) | - Direct costs: doctor visits, medication use, traveling expenses<br>- Indirect costs: informal care, parents´ loss of (non) paid work, tutoring of adolescents<br><br>Source: cost diaries (parents) | Societal: €-406<br><br>Healthcare: €147 | Subjective sleep efficiency, Recovery (SE ≥ 85%) | €12,572 SW | 95%, €12,000 | EQ-5D | N.R. SE | 95%, 70%[f], N.R. | N.A. | Guided iCBT vs. gCBT:<br><br>ICER: £13,153 /recovered person iCBT produced less effect at less costs.<br><br>iCBT generated slightly more QALY at less costs. |

| Author | Cost categories | Δ Costs | Outcome | ICER | CEA | QALY | ICUR | CUA | CBA | Results |
|---|---|---|---|---|---|---|---|---|---|---|
| Thiart, 2016<br><br>Buntrock, 2021<br><br>Employer, Societal, Healthcare (2013, €) | - Direct costs: healthcare intervention<br>- Indirect costs: productivity losses<br><br>Source: TiC-P | Employer: €-418<br><br>Societal: €-991<br><br>Healthcare: €203 | Positive treatment response (ISI)*[k]<br><br>Symptom-free status (ISI)*[m] | Employer: €-1,512 SE<br><br>Societal: N.R. SE<br><br>Healthcare: €650 | Employer: 87%, N.A., €761<br><br>Societal: 94%, N.A., €1000<br><br>Healthcare: 6%, €500, €1500 | SF-6D*[m] | Societal: N.R. SE<br><br>Healthcare: €11,285 | Societal: 94%, 98%, 99%<br><br>Healthcare: 4%, 95%, 99% | NB: €417<br><br>BCR: €3.1 | Guided iCBT vs. WLC:<br><br>Employer:<br>ICER: £-1,583/responder<br>iCBT dominated WLC and produced a net benefit of £437 and a BCR of £3.2.<br><br>Societal:<br>iCBT dominated WLC generating more effect and QALY at less costs.<br><br>Healthcare:<br>ICER: £681/symptom-free status<br>ICER: £11,819/QALY gained<br>iCBT generated more effect and QALY at higher costs. |
| Ebert, 2018<br><br>Kählke, 2019<br><br>Employer Societal, (2013, €) | - Direct costs: health service uptake, patients' out-of-pocket costs, intervention costs<br>- Indirect cost: productivity losses<br><br>Source: TiC-P | Employer: €-189<br><br>Societal: €-380 | Symptom-free status by Jacobson and Truax**[d] | Employer: €-521 SE<br><br>Societal: €-1,063 SE | Employer: 67%, N.A., €1.500[f]<br><br>Societal: 70%, N.A., €2.500[f] | SF-6D | Societal: N.R. SE | Societal: 71%[f], 76%[f],79%[f] | NB €181<br>BCR €1.6 | Guided iMA vs. WLC:<br><br>ICER: £-546 (employer), £-1,113 (societal)/symptom-free person<br><br>iMA generated higher effects and QALY gained at lower costs, and a net benefit of £190 and a BCR of 1.06. |
| Lindsäter, 2019<br><br>Societal, Healthcare (2016, US$) | - Direct cost: healthcare, intervention<br>-Indirect costs: productivity losses<br><br>Source: TiC-P | Societal: $-77.24<br><br>Healthcare: $171.12 | Remission rate on PSS (reliable change, post-rating PSS<31)**[d] | Societal: $-158 SE<br><br>Healthcare: $349 NE | Societal: 60%, N.A., $1000<br><br>Healthcare: 12%[f], $400[f], $1000[f] | EQ-5D-3L | Societal: $-17,963 SE | Societal: 60%, 71%[f], 75%[f] | N.A. | Guided iCBT vs. WLC:<br><br>Societal:<br>ICER: £-115/responder<br>ICUR: $-13,109/QALY gained<br>iCBT generated more effect and QALY at less costs.<br><br>Healthcare:<br>ICER: £-255<br>iCBT generated higher effects and costs. |

| Author | Cost categories | Δ Costs | Outcome | ICER | CEA | QALY | ICUR | CUA | CBA | Results |
|---|---|---|---|---|---|---|---|---|---|---|
| Van Spijker, 2012<br><br>Societal (2009, €) | - Direct cost: health service uptake, patients' out-of-pocket costs, intervention, medication<br>- Indirect cost: productivity losses<br><br>Source: TiC-P | €-5039 (annualised costs) | Treatment response (improvement of 6.48 on BSS)*[n] | €-34,727 SE | 91.5%, N.A., €18,000[f] | N.A. | N.A. | N.A. | N.A. | Unguided iCBT vs. WLC+:<br><br>ICER: £-39,753/responder<br><br>iCBT generated less costs per responder. |

Note:
*AC* Attention control group, *AD-SUS* Adult Service Use Schedule, *BDI* Beck's Depression Inventory, *BSS* Beck Scale for Suicide Ideation, *CBA* Cost-benefit analysis, *CG* control group, *CEA* Cost-effectiveness analyses, *CEAC* cost-effectiveness acceptability curve, *CES-D* Center of Epidemiologic Studies Depression Scale, *CORE-OM* Clinical Outcomes in Routine Evaluation–Outcome Measure; *CUA*, Cost-utility analyses; *CSSRI*, Client Sociodemographic and Service Receipt Inventory, *CY-BOCS* Children's Yale-brown obsessive compulsive scale, *Δ Costs* Mean incremental costs per participant (intervention group minus control group), *EQ-5D-3L/5L* European quality of life index version 5D with 3 or 5 level, *F2F* Face-to-face, *FU* Follow-up, *gCBT* Group-administered cognitive-behavioural therapy, *HAI* Health Anxiety Inventory, *iCBT* internet-based cognitive behavioural therapy, *ICD* International Classification of Diseases, *ICER* incremental cost-effectiveness ratio, *ICUR* incremental cost-utility ratio, *IG* intervention group, *iMA* internet-based interventions applying mixed approaches, *iPPI* internet-based Positive Psychology Intervention, *iPST* internet-based problem-solving therapy, *iPCT* internet-based preventive cognitive therapy, *ISI* Insomnia Severity Index, *LSAS* Liebowitz Social Anxiety Scale, *Max.* maximal probability of cost-effectiveness, *N.A.* not applicable, *N.R.* not reported; *p* probability of a treatment being cost-effective when compared with a control condition**,** *PDS-5* Posttraumatic Diagnostic Scale for DSM-5, *PSSRU* Personal Social Services Research Unit, *PDSS* Panic Disorder Severity Scale, *PHQ-9* Patient Health Questionnaire-9, *PRODISQ* PROductivity and DISease Questionnaire, *PSS-10* Perceived Stress Scale, *SC* Standard Care, *SCP* Stepped care pathway, *SE* Subjective sleep efficiency, *SF-6D* Short From six dimensions based on SF-36 Health Survey, *SPIN-17* Social Phobia Inventory, *TAU* Treatment as usual, *TAU*+ Treatment as usual with additional information e.g. web-based psychoeducation, *TDABC* Time-driven activity-based costing method, *TiC-P* Trimbos/iMTA Questionnaire on Costs Associated with Psychiatric Illness, *uGPC* Usual General Practitioner Care, *Vs.* versus, *WHO HPQ* World Health Organization Health and Work Performance Questionnaire, *WLC* Wait list control condition, *WLC+* WLC with additional information during the waiting period, *Y-BOCS* Yale-Brown Obsessive-Compulsive Scale, *AU$* Australian Dollar, *€* Euro, *£* Pound Sterling, *US$* USDollar

The significance level *$p<0.05$, ** $p<0.001$ for health-related outcomes and QALY are stated, if reported
Cost-benefit analysis was interpreted using this formula: CBA = NB>0, BCR>1, and ROI>0% = financial returns

[a] According to the location of the point estimate in the incremental cost-effectiveness plane the abbreviation South-East (SE), North-East (NE), South-West (SW), and North-West (NW), was used to indicate +/- cost and effects of the intervention compared to the control condition: SE ICERs are located in the south-east quadrant meaning that the intervention is more effective at lower costs (dominant); NE ICERs are located in the north-east quadrant meaning that the intervention is more effective at higher cost; NW ICERs are located in the north-west quadrant meaning that the intervention is less effective at higher cost (dominated); SW ICERs are located in the south-west quadrant meaning that the intervention is less effective at lower cost
[b] The WTP given certain probabilities of CE (e.g., probability of CE if the WTP is zero, *P* (WTP = 0) based on the CEAC are reported. The probabilities of the CE given certain WTP thresholds (e.g., for a WTP of £20,000, there is a 95% intervention's probability of being cost-effective, WTP ($p$ = 0.95) based on the CEAC are reported.
[c] All outcomes were indexed to a 2020 reference year and converted to Pound Sterling (£) using purchasing power parities (PPPs)
[d] The $\chi^2$ test was used for statistical analysis
[e] The independent *t*-test was used for statistical analysis
[f] The probability of the ICER given a specific WTP threshold or the WTP according to a given probability was not stated, thus the value was estimated based on the CEAC

[g] One-way ANOVAs (analysis of covariance) were used for statistical analysis
[h] A linear mixed model with restricted maximum-likelihood estimation (REML) and an unstructured (UN) covariance structure was used for statistical analysis
[i] Mixed-effects model analyses were used for statistical analysis
[j] A logistic regression model was used for statistical analysis
[k] A linear regression model was used for statistical analysis
[l] The Fisher's exact test was used for statistical analysis
[m] A bootstrapped seemingly unrelated regression equation model (bias-corrected accelerated 95% Cis) was used for statistical analysis
[n] A linear probability model considering the clustered data structure was used for statistical analysis
[Nb] The net-benefit approach was used for the analysis (net-benefit regression framework)

**Supplementary table 2: Medical subject headings (MeSH) used for searching the Medline data base**.

| Building blocks | Treatment | Disorder | Delivery mode | Costs | Randomized trial |
|---|---|---|---|---|---|
| MEDLINE | "Psychotherapy"[mh] OR Psychotherap* OR Therap* OR Intervention OR Treatment OR Prevent* OR prevention | "Depression"[mh] OR depress* OR mood OR Bipolar OR Dysthymi* OR mania* OR anxi* OR Stress* OR Phobi* OR Panic* OR agoraphobi* OR social anxi* OR generali* anxi* OR GAD OR OCD OR "Obsessive–compulsive disorder"[mh] OR PTSD OR post-traumatic stress OR health anxiety OR hypochondria OR self-harm OR suici* OR sleep* OR insomnia | "Computer Assisted Therapy"[mh] OR Internet[mh] OR "Computer-Assisted Instruction"[mh] OR Online OR Online based OR Web OR Web based OR Internet OR Internet based OR World wide web OR Computerized OR computer | "Costs and Cost Analysis"[mh] OR "Cost-Benefit Analysis"[mh] OR "Healthcare Costs"[mh] OR "Cost of Illness"[mh] OR "Quality-Adjusted Life Years"[mh] OR Economic OR Cost utility OR Health economic OR Cost* | Efficacy OR "Randomized Controlled Trials as Topic"[mh] OR "Randomized Controlled Trial"[mh] OR Randomized controlled trial OR Randomized trial OR RCT |
